# Psychiatric and Neurological Involvement in COVID-19 Hospitalized Patients Through the Global Pandemic in Central Romania

**DOI:** 10.3390/jcm15083030

**Published:** 2026-04-16

**Authors:** Claudia Daniela Lupu, Vlad-Dan Cotuțiu, Victoria Birlutiu

**Affiliations:** 1 Infectious Diseases Department, Academic Emergency Hospital Sibiu, 550245 Sibiu, Romania; victoria.birlutiu@ulbsibiu.ro; 2 Department of Parasitology and Parasitic Diseases, University of Agricultural Sciences and Veterinary, Medicine of Cluj-Napoca, Calea Mănăstur 3-5, 400372 Cluj-Napoca, Romania; vlad.cotutiu@usamvcluj.ro; 3 AINIMAL SRL, Strada Stejarului 41D, 407280 Florești, Romania; 4 Faculty of Medicine, Lucian Blaga University of Sibiu, 550169 Sibiu, Romania

**Keywords:** COVID-19, neuropsychiatric manifestations, pandemic waves, neurological complications, psychiatric disorders, hospitalized patients

## Abstract

**Background:** Neuropsychiatric manifestations are a recognized complication of COVID-19, yet their temporal evolution across pandemic waves remains poorly characterized in hospitalized cohorts. This study examined whether their prevalence and composition changed across five successive waves. **Methods:** We conducted a retrospective observational study of 1471 hospitalized adults with confirmed Severe acute respiratory syndrome coronavirus 2 (SARS-CoV-2) infection at Sibiu County Emergency Clinical Hospital, Romania (March 2020–January 2025), spanning ancestral through Omicron variants. A custom natural language processing pipeline extracted symptoms, medications, and International Classification of Diseases, 10th Revision (ICD-10) codes from electronic medical records. Nine hierarchical clinical clusters were defined; temporal trends were assessed using multivariable logistic regression with age-stratified replication. **Results:** Severe neurological presentations (stroke, seizures, hemiparesis) increased six-fold from 3.5% in Wave 1 to 20.1% in Wave 5, while psychiatric symptoms (anxiety, insomnia) declined from 13.3% to 4.3%. Overall, neuropsychiatric burden remained stable (~40–45%), revealing a compositional shift. This neurological trend persisted after multivariable adjustment (adjusted odds ratio 4.34, for Wave 5 vs. Wave 1) and within age-stratified subgroups, was inversely associated with respiratory severity and could not be attributed to vaccination status. The composite neurological severity index independently predicted mortality and intensive care unit admission. **Conclusions:** Neuropsychiatric manifestations in hospitalized Coronavirus disease of 2019 (COVID-19) patients underwent a compositional shift from psychiatric dominance in early waves to severe neurological dominance in later waves, consistent with a transition from reactive psychiatric presentations toward progressive neurological injury. This pattern, largely independent of measured confounders, underscores the need for sustained neurological surveillance beyond the acute respiratory phase.

## 1. Introduction

Severe acute respiratory syndrome coronavirus 2 (SARS-CoV-2), first identified in December 2019 in Wuhan, China [1], was declared a pandemic by the World Health Organization (WHO) in March 2020. By early 2023, over 760 million infections and 7.1 million deaths were recorded [2]. Initial reports centered on respiratory disease, while multi-organ involvement was later recognized [3,4]. Successive variants (Alpha, Delta, Omicron) differed in transmissibility and virulence [5,6]. Mass vaccination, from late 2020, reduced hospitalizations despite uneven uptake [7,8]. Phylogenetic evidence supports a zoonotic origin, with bat-borne RaTG13 sharing ~96% genome identity [9,10]. SARS-CoV-2 infects a broad host range: cats, ferrets, mink [11,12,13], hamsters [14], and non-human primates [15], producing neurological signs independent of respiratory severity. Neurotropism is conserved across beta coronaviruses: in mice, severe acute respiratory syndrome coronavirus 1 (SARS-CoV-1) invades the central nervous system (CNS) via the olfactory bulb [16], and Middle East respiratory syndrome coronavirus (MERS-CoV) causes encephalitis with cerebrospinal fluid viral detection [17,18].

Clinical management evolved rapidly: reverse transcription polymerase chain reaction (RT-PCR) established the diagnostic standard [19]; dexamethasone reduced mortality in oxygen-dependent patients [20]; remdesivir benefited selected groups [21]; and anticoagulation became routine following recognition of SARS-CoV-2-associated coagulopathy [22]. Together with growing population immunity, these advances lowered case-fatality rates, yet the elderly and immunocompromised patients remained at disproportionate risk [23]. Early pandemic case-fatality exceeded 5% globally (<1% to over 9% across countries) [24,25]. Delta (B.1.617.2, mid-2021) carried higher hospitalization risk than Alpha [6,26], whereas Omicron (B.1.1.529) caused milder individual disease yet unprecedented case volumes [27]. Secondary infections compounded severity. Bacterial pneumonia (3–14%) [28] and aspergillosis (up to 33% of ventilated patients) [29] worsened outcomes further.

Romania reported approximately 3.4 million confirmed infections and over 68,000 deaths by 2023 [30]. Only ~42% of the population completed the primary vaccination series by late 2022, the lowest rate in the EU [31], coinciding with Delta-period mortality peaks that exceeded ICU capacity in several counties, including Sibiu [32].

Against this backdrop, neurological involvement exceeded initial estimates. Mao et al. [33] reported neurological manifestations in 36.4% of 214 Wuhan inpatients, also corroborated by the ALBACOVID registry (57.4% of 841 patients [34]) and later by Jachman-Kapułka et al. [35] (55.4% of 426 Polish patients during the Omicron era), who documented delirium and encephalopathy increasing despite lower overall severity. Post-acute sequelae: fatigue (40–45%), cognitive complaints (25–30%) beyond three months [36] and stroke (1.4%) [37] compounded the burden. Proposed mechanisms include cytokine-driven neuroinflammation, blood–brain barrier disruption, angiotensin-converting enzyme 2 (ACE2)-mediated viral entry, and hypoxia-ischemia [12,38], with endothelial and cytokine-mediated pathways increasingly dominant in later waves [12,35,38,39].

Post-Coronavirus disease of 2019 (COVID-19) psychiatric morbidity is well documented in survivors: depression (14–17%), anxiety (13–23%), and sleep disturbances (<31%) [36,40,41]. Taquet et al. [42] found that mood and anxiety risks returned to baseline within months, whereas cognitive deficit, dementia, and epilepsy risks remained elevated throughout follow-up. Delirium affected 20–30% of hospitalized patients on supplemental oxygen and over 50% of those ventilated [43,44], compared with 10–15% in seasonal influenza [45]. The persistence of neurological sequelae beyond the acute phase is not unique to COVID-19. The 1918 influenza pandemic produced post-encephalitic Parkinsonism years after initial infection [46], and influenza-associated encephalopathy occurs independently of respiratory severity [47].

We therefore conducted a retrospective, wave-stratified analysis of 1471 consecutively hospitalized adults with confirmed SARS-CoV-2 at Sibiu County Emergency Clinical Hospital, Romania (March 2020–January 2025), aiming to (i) determine whether neuropsychiatric prevalence and composition changed across waves, (ii) distinguish psychiatric symptom clusters from severe neurological phenotypes, and (iii) assess documentation consistency through multi-tier data integration. To our knowledge, no single-center study has tracked neuropsychiatric manifestations across all five waves in an Eastern European setting where vaccination coverage remained below 42% (EU average ~73%) [31].

## 2. Materials and Methods

### 2.1. Study Design and Population

A retrospective cohort of 1471 adult patients was randomly sampled from all SARS-CoV-2–positive hospitalizations at Sibiu County Emergency Clinical Hospital between March 2020 and January 2025. Inclusion criteria were: (1) age ≥ 18 years at admission; (2) laboratory-confirmed SARS-CoV-2 infection (RT-PCR or commercially available rapid antigen test); (3) hospitalization duration > 1 day; and (4) primary or secondary diagnosis of COVID-19–related disease (International Classification of Diseases, 10th Revision [ICD-10]: J12.8 viral pneumonia and/or U07.1 confirmed SARS-CoV-2). Exclusion criteria were: (1) age < 18 years; (2) no laboratory-confirmed SARS-CoV-2 test; and (3) emergency department visit without subsequent inpatient admission. Readmissions of the same patient were included as separate episodes if they occurred during different pandemic waves, as each admission reflected distinct clinical and epidemiological circumstances. Pandemic waves were defined according to the predominant circulating SARS-CoV-2 variants in Romania, based on national surveillance reports and published clinical analyses. The cohort was stratified as follows: Wave 1 (Ancestral/Wild Type; March–September 2020, n = 345), Wave 2 (Alpha; October 2020–June 2021, n = 247), Wave 3 (Delta; July–December 2021, n = 274), Wave 4 (Omicron-early; January–June 2022, n = 282), and Wave 5 (Omicron-dominant period, including later sub-lineages; July 2022–January 2025, n = 323).

The total recorded COVID-19 hospitalizations at Sibiu County Emergency Clinical Hospital during the study period exceeded 5000, with a highly uneven wave distribution (>40% in W1). This wave distribution discrepancy between W1 and subsequent waves was attributable, in part, to patients who were admitted solely for confirmatory testing and immediately discharged for self-isolation during the first wave. From this pool, approximately 30% of eligible patients per wave were randomly selected via computer-generated random numbers, yielding approximately balanced but not identical wave subsamples (W1: n = 345, W2: n = 247, W3: n = 274, W4: n = 282, W5: n = 323). Residual differences in wave subgroup sizes reflect the available pool of eligible patients within each wave period. No formal a priori power calculation was performed, as this study analyzed the full available hospitalized cohort at this center using wave-stratified random sampling. At the achieved sample size (N = 1471), the minimum detectable effect at 80% power was Cramér’s V = 0.090, indicating adequate sensitivity for clinically meaningful associations and well below the observed primary effect (V = 0.255). However, age-stratified subgroup analyses within individual waves may be underpowered for small cell counts.

The study was conducted in accordance with the Declaration of Helsinki and was approved by the Institutional Ethics Commission, with annual renewal throughout the study period. Upon admission, all patients provided written informed consent in accordance with institutional regulations and the General Data Protection Regulation (GDPR). For the purpose of this retrospective study, patient data were analyzed in anonymized form. No additional study-specific informed consent was required.

### 2.2. Data Sources and Extraction

All data were extracted from electronic medical records (history, admission status, clinical evolution, specialty notes, discharge summaries, and administrative data) using a custom six-step Python 3.12 (Python Software Foundation, 2023, Beaverton, OR, USA) natural language processing (NLP) pipeline, building on established approaches for large-scale clinical phenotyping via NLP [48] and structured information extraction from clinical narratives [49]. Four expert-curated dictionaries encoded symptoms (91 items, 9 clusters with severity weights), medications (247 items), physical-examination findings (152 codes), and syndromes (24 items). ICD-10 codes (reference set of 71,704) were extracted for secondary-diagnosis enrichment. Extraction fidelity was verified by manual review of a representative subset (dictionaries available in Supplementary Material S1–S3).

Vaccination status was extracted from electronic discharge summaries using a custom NLP parser scanning structured diagnosis fields and free-text narratives (Romanian-language pattern matching with negation detection). A total of 1424/1471 patients (96.8%) had a corresponding discharge record. Binary vaccination status (vaccinated vs. unvaccinated) was derived for patients with documented COVID-19 vaccination mentions. Crude and wave- and age-adjusted logistic regression assessed vaccination–outcome associations for eight neuropsychiatric endpoints. Ordinary Least Squares (OLS) regression assessed vaccination–severity score associations for six continuous scores. Cochran–Mantel–Haenszel (CMH) testing stratified by wave confirmed the confounding structure. Full results are presented in Supplementary Table S32.

### 2.3. Neuropsychiatric Classification System

Symptoms were classified into nine hierarchical clinical clusters based on expert clinical judgment. Each symptom was assigned to exactly one cluster and received a severity weight reflecting its clinical acuity and impact on neuropsychiatric prognosis (Table 1).

For symptoms with inherent dual classification (NECA: syncope; NEPSY3: hallucinations), deduplication logic ensured each symptom was counted once per patient, using the maximum weighted value to avoid inflating cluster scores.

#### 2.3.1. Derived Indicators

From the nine cluster scores, binary presence indicators were computed: has_NEURO (any of NE2, NE3, NECA, or NEPSY3 positive), has_PSY (any of PSY1, PSY2, or NEPSY3 positive), and has_NEUROPSY (any neurological or psychiatric cluster positive). In addition, clinical-tier variables (has2_*) were independently derived from four clinical data sources—clinical evolution notes, discharge summaries, radiology reports, and specialty consultations—each parsed for findings in five clinical domains (CA, NE, PSY, RE, NC). Cross-source concordance variables quantified agreement between symptom-level and clinical-level detection.

#### 2.3.2. Modified Neurological Severity Index (mNSI)

The mNSI was constructed as a novel continuous composite metric:

mNSI = D_clin + D_spec

where D_clin is the neurological evolution score derived from longitudinal clinical notes, normalized by the cohort maximum (range 0–1). Whereas, D_spec is a composite specialty consultation score that aggregates the breadth and specificity of neurological findings across all relevant specialty assessments, including a binary neurology-referral indicator, the count of specific neurological diagnoses confirmed by consulting specialists, and the severity of cerebral findings documented in specialty reports, normalized to 0–2. The resulting index ranges from 0 to 3 (observed maximum 2.5). The index’s prognostic performance was assessed against in-hospital mortality (Mann–Whitney U) and ICU admission, with its incremental predictive value assessed via likelihood-ratio tests.

### 2.4. Statistical Analysis

All statistical analyses were implemented in Python 3.12 using the following packages: statsmodels (v0.14.6), scipy.stats (v1.17.0), scikit-learn (v1.8.0), and scikit-posthocs (v0.12.0). Model assumptions, multi-collinearity (variance inflation factor), and convergence diagnostics were evaluated for all multivariate analyses. The pipeline consisted of 21 phases organized into core analyses (Phases 2–10) and supplementary analyses (B1–B6). A complete inventory of all statistical tests and methods employed, together with where each was applied and the rationale for its selection, is provided in Table S12.

#### 2.4.1. Incidence Analysis

Wave-specific prevalence of neuropsychiatric outcomes was quantified using Pearson’s χ^2^ tests for overall heterogeneity (Fisher’s exact test where expected cell counts fell below 5) and Cochran–Armitage tests for linear trend across ordered wave categories. Post hoc pairwise comparisons employed Fisher’s exact test for all 10 wave pairs, with Benjamini–Hochberg (BH) correction at α = 0.05 to control the false discovery rate. Effect sizes were reported as Cramér’s V and odds ratios with 95% confidence intervals (reference category: Wave 1). These analyses were conducted across three tiers—symptom-level (11 variables), clinical-level (26 variables from 4 sources × 5 domains plus 6 aggregates), and cross-source concordance (6 variables)—and replicated within ICU, non-ICU, and oxygen-stratified subgroups. Age-stratified models (18–40, 41–60, 61–75, ≥76, and non-geriatric group 18–75) tested the robustness of the temporal divergence across demographic strata.

#### 2.4.2. Multivariate Modelling

Maximum-likelihood binary logistic regression evaluated wave-dependent trends while adjusting for age, hospitalization duration, secondary diagnosis burden, ICU admission, supplemental oxygen, complication count, and clinical resolution (N = 708 with complete covariate data). Sex was assessed in dedicated stratified and interaction analyses and excluded from the primary multivariate models given its null association with the core NE3 outcome (OR = 1.285, *p* = 0.21). Clinical resolution was collapsed from five original categories into three (cured, improved, deceased) to avoid sparse cells. Model diagnostics comprised variance inflation factors, likelihood-ratio comparisons of nested models (wave-only vs. full), McFadden’s pseudo-R^2^, and Hosmer–Lemeshow goodness-of-fit tests. Three covariates exceeded the VIF > 5 threshold: age (13.82), secondary diagnosis count (8.69), and hospitalization days (5.39), consistent with known age-related intercorrelation. All three were retained as conceptually distinct confounders, as elevated VIF inflates standard errors without biasing point estimates. All 11 primary models converged successfully using Newton’s method (full coefficients in Table S6; VIF values in Table S7). Interaction extensions assessed sex moderation (44 wave × covariate interaction terms), ICD-10 chapter effects (J-respiratory vs. non-J), seasonal/daylight influences using daylight hours as a continuous proxy computed from solar declination at the study latitude (45.78° N), and respiratory–neurological mediation via Baron–Kenny three-step testing percentile bootstrap confidence intervals (5000 resamples).

#### 2.4.3. Detection-Validity Analyses

Cases were classified by detection method: symptom-only, medication-confirmed, specialty-confirmed, and triple-confirmed (symptom + medication + specialty). Temporal shifts in ascertainment proportions were tested with Fisher’s exact tests. Concordance between anti-epileptic medication use and epilepsy-specialty consultation was evaluated to assess alignment between prescribing patterns and specialist-documented diagnosis. Specialty consultation patterns, radiological cerebral involvement, and daylight–age interactions were modeled using logistic and ordinary least-squares regression, with effect estimates interpreted as non-causal associations.

#### 2.4.4. Mechanistic and Clinical Linkage Analyses

The modified Neurological Severity Index (mNSI) was validated using non-parametric tests and likelihood-ratio incremental models. Attenuation of wave effects was estimated after adjusting for neurological comorbidity burden and phenotype-specific components (cerebrovascular, motor deficit, neurodegenerative, psychiatric primary, behavioral), with percent attenuation calculated as (OR_base − OR_adjusted)/(OR_base − 1) × 100. Observational treatment associations were evaluated through odds ratios by medication class, corticosteroid × O_2_ interaction terms, and wave-trend χ^2^ tests, with the non-causal nature of discharge-level medication data explicitly acknowledged.

#### 2.4.5. Integrated Prediction Modelling

Backward-elimination logistic regression with sequential domain-block entry (demographics → clinical severity → wave → symptom clusters → clinical data sources) was applied to neuropsychiatric detection and mortality outcomes (Table S18). For each block, incremental contributions were evaluated comparatively via ΔR^2^ (McFadden) and likelihood-ratio tests. Model performance was assessed using internal 5-fold stratified cross-validation (AUC, Brier score, calibration slope), with parallel comparisons of symptom-level versus clinical-level predictor sets (Table S16).

#### 2.4.6. Sensitivity and Ancillary Analyses, Secondary Diagnosis Enrichment by Cluster

Six supplementary analyses provided robustness and construct-validation checks. Seasonality was tested via χ^2^ tests and daylight correlations. Severity–neuropsychiatric linkage was assessed using Fisher’s exact tests, odds ratios, and wave × ICU interactions. Radiology–specialty convergence was evaluated using φ-coefficient matrices and Spearman correlations of radiological cerebral burden with clinical neurological scores. Cross-source concordance was quantified using Cohen’s κ with sensitivity/specificity estimation and κ trajectory across waves to assess diagnostic practice evolution. Exploratory principal-component–derived composite severity indices (CNSI) were constructed with hierarchical clustering (Ward’s method) and age × wave interaction testing. Cluster-level secondary diagnosis enrichment was assessed using Fisher’s exact tests with Benjamini–Hochberg correction applied per cluster (30 tests each) across 14 cluster definitions, with ICD-10 chapter profiling of 1553 unique codes from 20,930 code instances (Table 2).

## 3. Results

A total of 1471 hospitalized COVID-19 patients (M/F ratio of 0.83; mean age 63.6 years) admitted at Sibiu County Emergency Clinical Hospital across five pandemic waves (March 2020–January 2025) were included. The cohort aged significantly across waves, from a mean of 59.4 (W1) to 70.9 (W5) (*p* < 0.001). Hospitalization duration decreased from a mean of 15.6 days (W1) to 8.3 days (W5) (*p* < 0.001). Overall, 13.2% of patients required intensive care, 32.4% received supplemental oxygen, and 8.9% died during admission. ICU admission, in-hospital mortality, and sex ratio did not vary across waves (Table 3).

**Figure 1:**
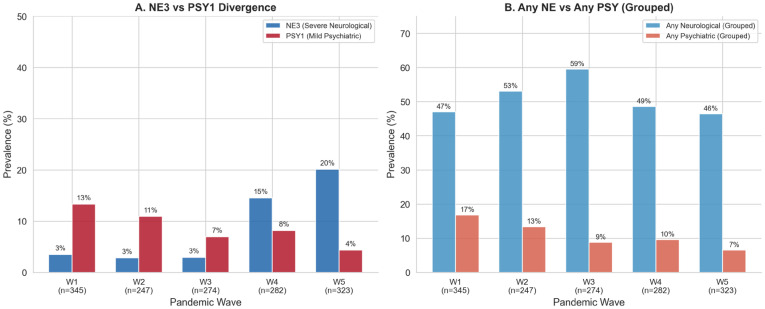
Neurological–Psychiatric Divergence Across Pandemic Waves (N = 1471). Panel (**A**): NE3 (severe neurological) vs. PSY1 (mild psychiatric) prevalence by wave. Panel (**B**): Grouped neurological vs. psychiatric prevalence at broader symptom thresholds. Global χ^2^ tests indicated statistically significant differences across waves (NE3: χ^2^ = 95.6, *p* = 8.3 × 10^−20^; PSY1: χ^2^ = 19.7, *p* = 5.9 × 10^−4^).

Neuropsychiatric involvement was documented in 521 patients (35.4%) and showed a marked divergence: severe neurological diagnoses (NE3) increased sharply across waves while psychiatric symptoms (PSY1) declined, producing the strongest trend signal in the study (Cochran–Armitage Z = 8.68, *p* < 10^−17^). This pattern was confirmed at symptom (ANY_PSY decline by 10.3%), clinical (has2_ANY_NE increase of 17.3%; has2_ANY_PSY decrease 7.9%), and cross-source tiers (NE rose by 14.7%, while PSY agreement fell by 9.8%). (Table 4, Figure 1).

### 3.1. Temporal Divergence Between PSY Decline and NE3 Increase

#### 3.1.1. Opposing Temporal Trajectories

PSY1 prevalence declined monotonically across waves (9% decrease) while NE3 followed the opposite trajectory (16.6% increase) (Table 4). The broader psychiatric grouping (ANY_PSY) and discharge-documented psychiatric findings (DISC_PSY) followed the same declining pattern (Table S1). Specialty neurological consultations (SPEC_NE) and cross-source concordance confirmed both trends: neurological agreement rose while psychiatric agreement fell across waves (Figure 1 and Figures S1–S4; Tables S3 and S4).

**Figure 2:**
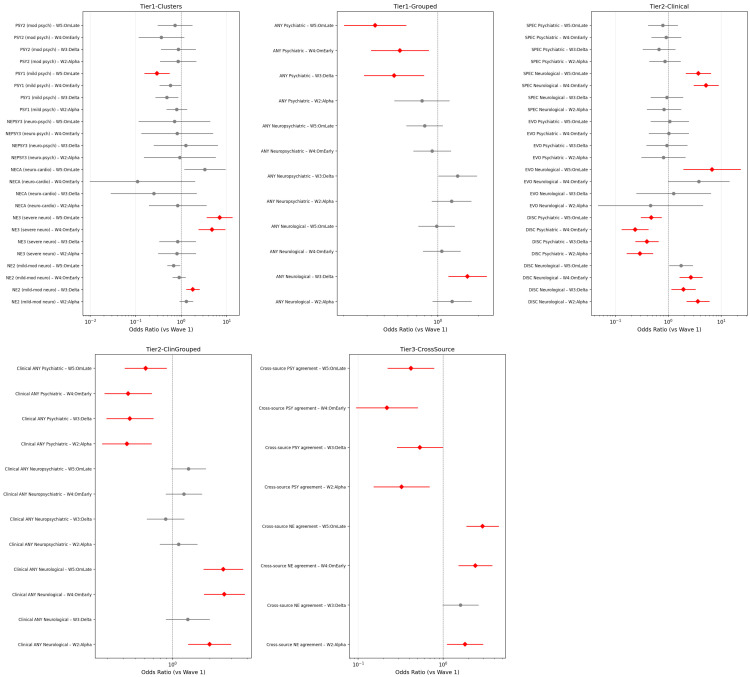
Forest plot of unadjusted odds ratios across all tiers (reference: Wave 1). Red (dot + line) = statistically significant after Benjamini-Hochberg FDR (BH-FDR) correction (*p* < 0.05); Gray (dot + line) = not statistically significant after BH-FDR correction.

Relative to Wave 1, unadjusted odds ratios showed 5- to 7-fold increased NE3 risk in W4–W5 and a 2- to 6-fold decrease in PSY1 across later waves, with clinical-tier variables confirming both directions (Figure 2; Table S2). Vaccination status did not confound these trends.

Age-stratified analyses replicated the core NE3–PSY divergence within five demographic subgroups (Table S31). The NE3 increasing trend was significant in non-geriatric (<61 years), middle-aged (45–64), elderly (≥65), and geriatric (≥61) strata. In the youngest stratum (<45 years), the NE3 trend was directionally consistent, but did not reach significance after BH correction, likely reflecting limited statistical power. The PSY1 decline was similarly consistent across all strata.

#### 3.1.2. Multivariate Analysis

**Figure 3:**
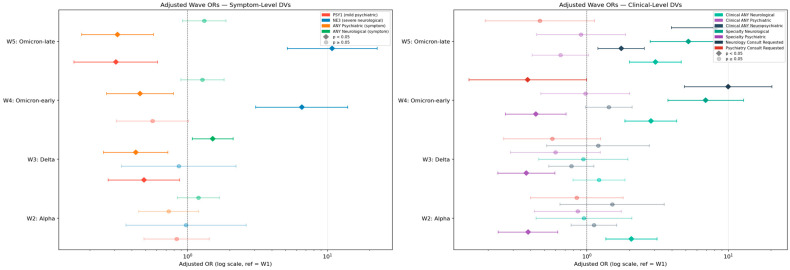
Adjusted forest plot—logistic regression ORs by wave adjusted for age, hospitalization days, comorbidity count, severity, and resolution (reference: Wave 1).

Logistic regression adjusting for seven covariates confirmed both wave trends after controlling for age, hospitalization duration, comorbidity load, ICU, O_2_ support, complication count, and discharge resolution (N = 708; all models converged; Hosmer–Lemeshow *p* > 0.05 for all; Table 4). Wave was the dominant predictor block for both NE3 (Δχ^2^ = 39.2, *p* = 6.3 × 10^−8^) and PSY1 (Δχ^2^ = 51.8, *p* = 1.6 × 10^−10^). Age and hospitalization days reached independent significance for NE3 but not PSY1, where wave effects accounted for the predominant model variance (Figure 3 and Figure S5; Tables S5–S8).

Broader clinical categories confirmed the directional pattern, with effects concentrated in Wave 4. The severity covariate block reached significance for ANY_PSY (Δχ^2^ = 15.9, *p* = 0.001), driven by ICU admission (Tables S5–S8).

#### 3.1.3. Aggregate Neurological Patterns

Aggregate neurological prevalence (ANY_NEURO, 50.5%) showed a statistically significant but small wave effect (V = 0.097), while moderate neurological burden (NE2) actually decreased across waves (V = 0.162). The divergence is thus compositional: moderate neurological presentations waned while severe presentations (NE3) rose within a stable aggregate envelope. Minor classifications (NECA, PSY2, NEPSY3) were rare and showed no meaningful temporal variation (Tables S1 and S26; Figure S2).

### 3.2. Systematic Confound Elimination

#### 3.2.1. Sex

Sex was a significant predictor for psychiatric outcomes (PSY1 sex OR = 0.546, *p* = 0.003) but did not confound the NE3 wave trend (OR = 1.285, *p* = 0.21). Of wave × sex interaction tests, only one reached significance (has2_SPEC_NE, *p* = 0.023), confirming that the NE3 finding is sex-unconfounded (Figure S6; Table S9).

#### 3.2.2. Seasonality

Seasonal variation reached significance for several neuropsychiatric outcomes (NE3 × season *p* = 0.034; NEURO × season χ^2^ = 18.63, *p* = 0.0003), but strong wave–season collinearity (χ^2^ = 404.7) precluded independent interpretation. Daylight duration as a continuous seasonal proxy showed minimal associations (3/18 significant, all ORs ≈ 0.95) (Table S22; Figures S8, S9, S12, S17 and S18).

#### 3.2.3. Oxygen Support and Clinical Respiratory Burden

The wave → O_2_ mediation path was null (*p* = 0.44; R^2^ ≈ 0.0), and bootstrap-based indirect-effect tests (5000 resamples) were non-significant for all outcomes: ANY_NE 95% CI [−0.008, 0.003], ANY_PSY [−0.012, 0.005], ANY_NEUROPSY [−0.011, 0.005]; attenuation −2.2% to −6.4%, indicating suppression rather than mediation. Clinical respiratory severity showed an inverse association with NE3 (OR = 0.704, *p* = 0.0001), opposite to a respiratory-mediation hypothesis, while positively associated with ANY_PSY (OR = 1.672, *p* < 0.0001) (Tables S10 and S11; Figures S10 and S11).

#### 3.2.4. Neuro-Comorbidity Burden

Pre-existing neurological comorbidity attenuated the NE3 wave effect by 23.2%, leaving 76.8% intact. Neurodegenerative attenuation was negligible (−0.1%), while cerebrovascular and motor-deficit phenotypes increased in parallel with NE3 and psychiatric phenotype decreased in parallel with PSY1. Specialty consultation count remained a strong independent predictor for both neurological and psychiatric detection (Table S13; Figures S13 and S14).

#### 3.2.5. Vaccination Status

Vaccination status was ascertained for 706 (49.6%) of patients, with the vaccinated proportion rising from 14.5% (W2) to 62.4% (W5). Vaccinated patients showed higher unadjusted NE3 prevalence (14.9% vs. 8.2%, *p* = 0.008), but this was entirely attributable to wave-level confounding: adjusted OR = 1.11 (*p* = 0.70); MH OR = 0.97 (*p* = 0.99). No neuropsychiatric outcome showed a significant vaccination association after adjustment (all adj *p* > 0.10) (Table S32).

#### 3.2.6. ICD-10 Chapter

J-chapter (Respiratory) diagnoses comprised 87.1% of the cohort. NE3 prevalence was lower among J-chapter patients (8.4%) than non-J patients (13.2%; OR = 0.608, *p* = 0.041), indicating that non-respiratory primary diagnoses carry higher NE3 odds (Tables S24 and S25).

#### 3.2.7. Wave × Covariate Interactions

Of 44 wave × covariate interaction terms tested (11 dependent variables × 4 interaction terms), 5 reached significance (Table S23; Figure S7).

#### 3.2.8. Confound Elimination Summary

None of the tested confounders explained the NE3 wave trend: sex was unrelated (*p* = 0.21); seasonality was inextricable from wave collinearity; oxygen mediation was null; and vaccination showed no association after wave adjustment. Pre-existing neurological comorbidity produced the largest attenuation (23.2%), but 76.8% of the wave effect persisted. Clinical respiratory severity was inversely associated with NE3, reinforcing rather than explaining the trend (Table 5).

### 3.3. Detection, Acuity, and Confirmation

#### 3.3.1. Medication Acuity

For NE3 cases (N = 133): 57.1% met criteria for suspected acute neurological events, and 19.5% achieved triple confirmation (symptom + medication + specialty). NE3 detection relied more on specialty consultation (51.1%) than medication confirmation (24.1%). Conversely, PSY1 cases (N = 129, 43.4% suspected acute) were more often medication-detected (38.0%) than specialty-referred (9.3%). Anti-epileptic medication showed 0% concordance with epilepsy specialty consultations, indicating that no specialist-documented epilepsy was present among AEM recipients.

#### 3.3.2. Cross-Source Concordance

Neurological concordance was low (κ = 0.153), largely reflecting the high proportion of symptom-only NE cases (n = 484). Psychiatric concordance was fair (κ = 0.327). NE κ trajectory increased across waves (W1 = 0.088 → W5 = 0.354), consistent with improved recognition and documentation of neurological presentations in later waves (NE sensitivity = 64.6%, specificity = 54.8%; PSY sensitivity = 35.0%, specificity = 93.5%).

#### 3.3.3. Consultation Cascade

Among patients with neurological symptoms, 14.5% received a neurology consult (χ^2^ = 78.0, V = 0.324). Among clinical NE+ patients, 24.7% received neurology follow-up at discharge. Among clinical PSY+ patients, 48.5% received psychiatric follow-up at discharge.

### 3.4. Clinical Characterization

#### 3.4.1. Modified Neurological Severity Index—mNSI

**Figure 4:**
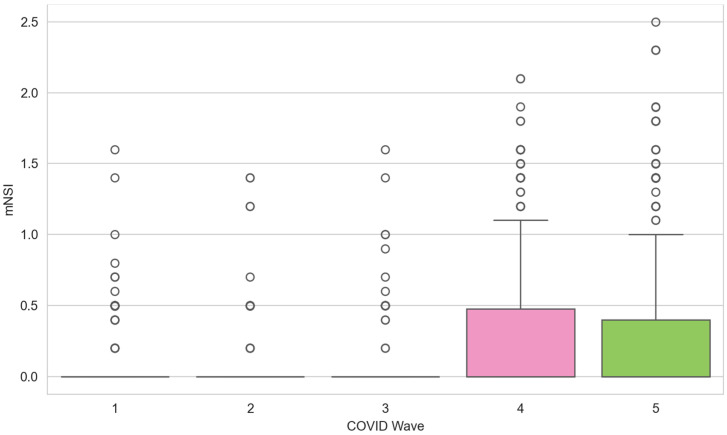
Modified Neurological Severity Index (mNSI) by pandemic wave.

**Figure 5:**
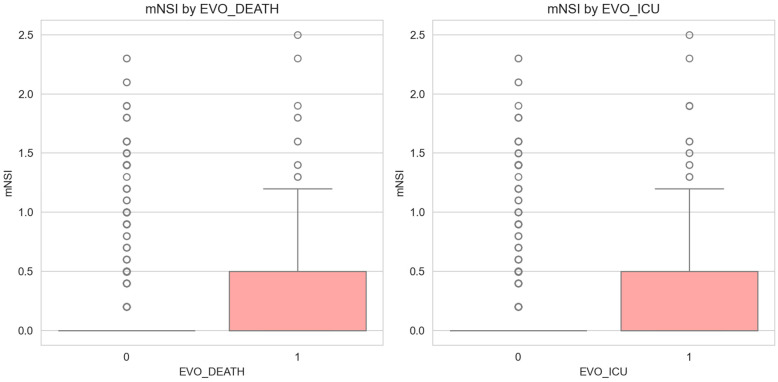
mNSI validation against mortality and ICU (Intensive Care Unit) outcomes.

The mNSI (mean = 0.146) was significantly associated with both in-hospital mortality (*p* = 5.1 × 10^−8^) and ICU admission (*p* = 1.5 × 10^−9^) and added independent predictive value beyond established clinical covariates (likelihood-ratio *p* = 0.010; OR = 1.658 per unit increase). Mean mNSI remained stable across W1–W3 (~0.05) before increasing approximately five-fold in the Omicron period (~0.28; Kruskal–Wallis *p* = 3.5 × 10^−26^), mirroring the NE3 prevalence rise (Figure 4 and Figure 5).

#### 3.4.2. Behavioral Disturbance

Behavioral disturbance events were near-tautologically associated with any psychiatric involvement (OR = 346.57). In unadjusted analysis, it was associated with a six-fold increase in mortality risk (OR = 5.94, *p* < 0.001), which was fully attenuated after multivariable adjustment (OR = 1.529, *p =* 0.32), suggesting confounding by severity-related covariates. Cross-source agitation concordance was high (OR = 18.44, *p* < 0.0001). Independent risk factors for behavioral disturbance were ICU admission (OR = 6.88, *p* < 10^−7^) and older age (OR = 1.03, *p* = 0.014), while pre-existing dementia was not a significant predictor, suggesting that behavioral disturbance is acute hospital-associated (Table S19).

### 3.5. Comorbidity and Treatment Associations

#### 3.5.1. Comorbidity Phenotype Trends

**Figure 6:**
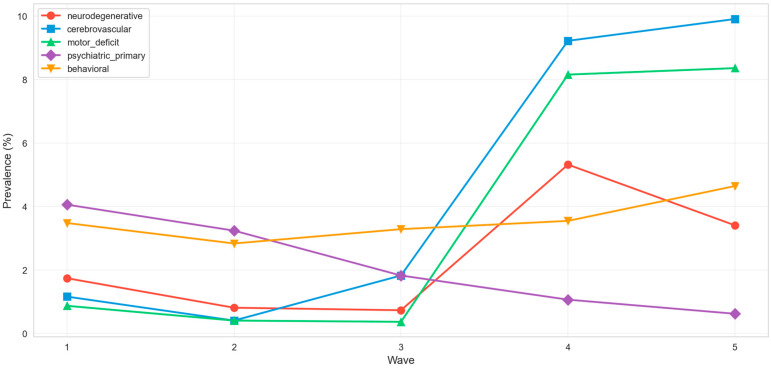
Comorbidity phenotype temporal trends by pandemic wave.

Comorbidity phenotype trends were directionally consistent with the NE–PSY divergence: cerebrovascular and motor deficit phenotypes showed an increasing trend (*p <* 0.001), neurodegenerative increased (*p =* 0.001), psychiatric primary decreased (*p =* 0.014), and behavioral showed no statistically significant variation (*p =* 0.82) (Figure 6).

#### 3.5.2. Treatment Effects

**Figure 7:**
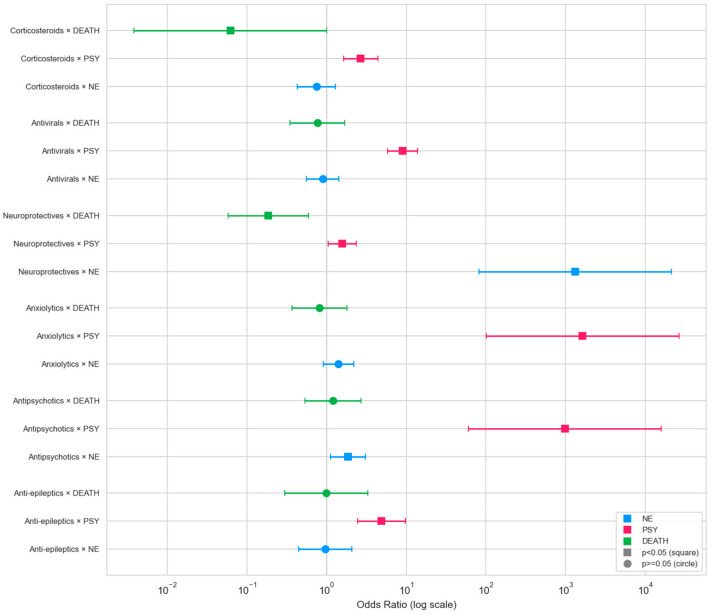
Forest plot of medication–neuropsychiatric outcome associations.

Corticosteroid use was associated with higher odds of any psychiatric outcome (ANY_PSY OR = 2.71, *p* = 0.0001), with no significant corticosteroid × O_2_ or corticosteroid × ICU interactions (Table S14; Figure S15). Neuropsychiatric medication prescribing declined across waves, from 19.1% (W1) to 12.4% (W5) (χ^2^ = 16.21, *p* = 0.003). ICU patients received significantly more anxiolytics (*p* = 0.011) and antipsychotics (*p* = 0.014) than non-ICU patients (Figure 7 and Figure S16; Tables S13 and S15).

### 3.6. Integrated Models

In the backward-elimination mortality model, significant predictors were age, wave, ICU admission, O_2_ support, and complication count (Table S17). Specialty consultation count (SPEC_N) was the single most informative predictor block for neurological detection, while behavioral events dominated psychiatric detection (Table 6). Symptom-level models outperformed clinical-level models for neurological outcomes (AUC = 0.86 vs. 0.76), whereas clinical-level models outperformed for psychiatric outcomes (AUC = 0.76 vs. 0.67).

### 3.7. Sensitivity and Ancillary Analyses

Several ancillary analyses explored temporal modifiers, severity linkages, and composite indices. Clinical severity markers confirmed that ICU admission was linked to both psychiatric (OR = 2.78, *p* = 2.5 × 10^−8^) and neurological outcomes (OR = 1.84, *p* = 0.0003), and stroke carried near-infinite odds for clinical NE and elevated mortality risk (OR = 7.20, *p* = 1.6 × 10^−4^) (Table S19). The Composite Neuropsychiatric Severity Index (CNSI) correlated with death (ρ = 0.43) and ICU admission (ρ = 0.59), yielding a mortality model R^2^ = 0.256. However, neither NE nor PSY independently predicted death after adjustment for O_2_ and ICU covariates (NE: OR = 1.35, *p* = 0.24; PSY: OR = 1.16, *p* = 0.59) (Table S19).

#### Secondary Diagnosis Enrichment by Cluster

NE3 showed the strongest enrichment pattern, with all 30 secondary diagnosis codes reaching significance. Top enrichments were cerebrovascular and motor-deficit codes (hemiplegia, dysphasia, stroke, cerebral infarction), while typical COVID accompaniments (headache, fever, fatigue) were depleted, confirming that NE3 as a stroke/motor-deficit phenotype dominated by ICD-10 chapters G and I (Table 2).

PSY showed a clean F-chapter (Mental/Behavioral) signature with no neurological code enrichments, supporting complete biological separation from the NE cluster. An unexpected finding was toxic liver disease enrichment (Table S30). SPEC_NE exceeded the NE3 pattern in severity, consistent with SPEC_N classification dominance (Table S18). ANY_PSY was predominantly psychiatric but included unexpected G-chapter enrichments (cerebrovascular syndrome, neurodegeneration), suggesting a subset with overlapping neurological pathology (Table S30).

## 4. Discussion

The present study examined the full spectrum of neuropsychiatric manifestations across all five pandemic waves (W1–W5, 2020–2025) in a consecutive cohort of 1471 hospitalized COVID-19 patients at Sibiu County Hospital, Romania. By spanning the entire pandemic timeline from the ancestral SARS-CoV-2 lineage through Alpha, Delta, and Omicron variants, this analysis captures temporal dynamics not assessable in cross-sectional or single-wave designs. Several principal findings emerged. Severe neurological presentations (NE3) increased approximately six-fold from W1 to W5, while psychiatric presentations (PSY1) declined by two-thirds. This divergence persisted after multivariable adjustment and proved compositional rather than absolute, as the aggregate neuropsychiatric burden remained stable across waves while its internal composition shifted decisively. ICD-10 enrichment analysis confirmed biological separation between clusters, with NE3 defined by a cerebrovascular-dominant diagnostic signature distinct from the symptom profile of typical COVID-19. The NE3 trajectory was inversely associated with respiratory severity, could not be attributed to vaccination status, and persisted within age-stratified subgroups, although residual age confounding cannot be fully excluded. A composite neurological severity index (mNSI) independently predicted mortality and ICU admission beyond established risk markers. These findings are consistent with a temporal transition from predominantly reactive psychiatric presentations in early waves, toward a progressive neurological pattern of injury in later phases. This pattern is examined through mechanistic, evolutionary, and methodological lenses before integration into a three-phase model.

Early pandemic syntheses [49,50] described neurological and psychiatric manifestations within a unified “neuropsychiatric” framework, reflecting the clinical landscape of the initial pandemic phase. In contrast, the present longitudinal wave-based analysis indicates that the relationship between neurological and psychiatric complications shifted across successive pandemic phases. This supports the interpretation of clinically distinct neurological and psychiatric trajectories evolving differently over time, consistent with separate pathophysiological substrates rather than a single neuropsychiatric continuum.

### 4.1. The NE3–PSY Temporal Divergence—Opposing Trajectories Across Pandemic Waves

The central finding of this study is the opposing temporal trajectory of severe neurological (NE3) and psychiatric (PSY1) clusters across successive pandemic waves. NE3 prevalence rose approximately six-fold from W1 to W5, an increase that remained statistically significant after multivariable adjustment for age, sex, oxygen support, ICU admission, and respiratory severity (Table 4; Figure 1, Figure 2 and Figure 3). Over the same interval, PSY1 declined by approximately two-thirds (Table 4). The following subsections characterize this divergence in detail, examine potential explanatory mechanisms, and situate the findings within cross-pathogen and experimental frameworks.

#### 4.1.1. Phenotypic Characterization and Prevalence Context

The aggregate neuropsychiatric rate remained stable across all five waves at both clinical and symptom-extraction tiers (Table 3), indicating that the overall burden of neuropsychiatric involvement did not change but its internal composition shifted decisively from psychiatric toward neurological dominance. This finding highlights that divergence is not simply an artefact generated by changing detection practices. A parallel redistribution occurred within the neurological category itself, as moderate presentations (NE2, characterized by headache, anosmia, and fatigue) gave way to severe phenotypes (NE3, including stroke, hemiplegia, dysphasia, and motor deficits) in later waves (Figure 5). ICD-10 enrichment analysis confirmed this pattern at the diagnostic level: NE3 was strongly enriched for cerebrovascular codes and depleted for common COVID-associated symptoms, while NE2 retained a symptom-dominant profile (Table 4; Tables S19 and S20). The concurrent depletion in NE3 patients suggests that this cluster represents a structurally distinct clinical entity rather than a typical disease course complicated by incidental neurological findings. This is consistent with trajectories observed in Omicron-era cohorts where cerebrovascular events persisted despite milder respiratory disease [35]. From a pathophysiological perspective, this shift from transient inflammatory symptoms toward lasting cerebrovascular injury is consistent with a transition from predominantly cytokine-driven mechanisms in early waves to endothelial and microvascular damage in later phases.

These findings warrant contextualization against the evolving body of COVID-19 neurology literature. The earliest descriptions of neurological involvement reported uniformly high prevalence during the first wave: Mao et al. [33] identified manifestations in 36.4% of 214 Wuhan inpatients, Helms et al. [51] documented neurological signs in 84% of 58 Strasbourg ICU patients, and Varatharaj et al. [52] recorded cerebrovascular events in 62% of 153 UK surveillance cases. None of these studies, however, had the longitudinal scope to determine whether neurological involvement changed in character over subsequent waves. Our W1 NE3 rate was considerably lower, most likely reflecting the broader severity spectrum of our unselected hospitalized cohort compared with the ICU-dominant samples in these early reports.

Large-scale electronic health record analyses provide a complementary perspective. Taquet et al. [41] (N = 236,379) reported a 6-month incidence of 33.6% for any neurological or psychiatric diagnosis, while their extended follow-up [42] (N = 1,284,437) showed that psychiatric risks tended to normalize over two years, whereas neurological risks, particularly cognitive deficit, dementia, and epilepsy, remained persistently elevated. Although these studies focused on post-acute outcomes in predominantly outpatient populations, the pattern of attenuating psychiatric risk alongside sustained neurological vulnerability conceptually mirrors our acute in-hospital findings. This convergence across acute and post-acute settings suggests that the neurological trajectory identified during hospitalization may represent the early phase of a longer-term process with implications for follow-up planning and rehabilitation resource allocation.

#### 4.1.2. Variant-Specific Mechanisms and the Role of Vaccination

The rise in NE3 during later waves, dominated by Delta and Omicron variants, appears paradoxical given reports of reduced systemic severity with Omicron [6]. Neurotropism, however, may operate at least partially independent of respiratory severity.

Douaud et al. [53] demonstrated structural brain changes in patients with largely mild disease, and Bowe et al. [54] showed that reinfection was associated with cumulative neurological risk. Tang et al. [55] used serial MRI in hamsters to demonstrate that the ancestral WH-09 strain and the immune-evasive XBB.1 sub variant produced extensive olfactory bulb and limbic inflammation, whereas BA.1 and BF.7 Omicron sub variants caused markedly attenuated neuroinflammation. This variant-specific attenuation of olfactory and limbic pathology [55] may explain why psychiatric trajectories resolved more readily, while cerebrovascular endothelial injury persisted or accumulated through repeated exposure [39,54]. Clinically, these findings imply that the neurological risk profile of SARS-CoV-2 cannot be inferred from its respiratory phenotype alone, and that repeated infections may compound cerebrovascular vulnerability even when individual episodes are mild.

Complementary meta-analytic and cohort data [35,37,56] corroborate persistent neurological complications despite milder respiratory disease, consistent with the dissociation observed in our cohort. Early COVID-19 neurology literature described a spectrum from mild symptoms to major cerebrovascular events: Liotta et al. [57] reported predominantly mild manifestations corresponding to our NE2 phenotype, while stroke-focused studies [58,59] documented severe complications analogous to NE3. Paterson et al. [60] identified heterogeneous neurological categories in UK patients, but their cross-sectional design precluded the temporal comparisons that our wave-stratified analysis enables. The population-level shift across successive waves has not been captured in prior designs and may carry implications for how neurological risk is stratified in future pandemic preparedness frameworks.

The role of vaccination as a potential modifier of neuropsychiatric outcomes warrants consideration, given the temporal overlap between vaccine rollout and the observed neurological escalation. Vaccination status was available for approximately half of the cohort, with expected temporal patterns: predominantly unvaccinated in earlier waves and increasingly vaccinated in later waves (Table S32). Crude analysis suggested higher NE3 prevalence among vaccinated patients, but this association was entirely attributable to confounding by pandemic wave, as vaccinated patients were concentrated in W4–W5, where both older demographics and evolving viral characteristics independently elevated NE3 risk. After adjustment for wave and age, vaccination showed no independent association with any neuropsychiatric outcome, a finding confirmed by Cochran–Mantel–Haenszel stratified analysis (Table S32). Vaccination was, however, associated with reduced pulmonary burden, consistent with attenuated respiratory severity in vaccinated individuals reported in large cohort studies [6]. These results suggest that COVID-19 vaccination primarily modified respiratory rather than neuropsychiatric disease severity in our hospitalized cohort, and that the temporal trends in NE3 and PSY1 are not artifacts of differential vaccination rates.

#### 4.1.3. Cross-Pathogen Parallels and Experimental Evidence

Since neither variant-specific respiratory attenuation nor vaccination status accounts for the NE3 trajectory, the question arises whether analogous patterns exist in other neurotropic infections. Cross-pathogen comparisons require cautious interpretation, yet the available evidence reveals a consistent theme: analogous divergence between neurological persistence and psychiatric attenuation has been described in other neurotropic infections. Post-infectious syndromes following influenza-associated encephalitis lethargica demonstrated that neurological sequelae can emerge or intensify years after initial infection, while acute psychiatric symptoms resolve earlier [46]. HIV-associated neurocognitive disorders evolved distinctly from psychiatric comorbidity across the combination antiretroviral therapy era, with cognitive decline persisting after adjustment for depression and substance use [61,62]. West Nile virus neuroinvasive disease follows a similar pattern, progressing from acute encephalitis to long-term motor and cognitive deficits while psychiatric presentations resolve [63,64]. Comparable dissociations between neurological and respiratory severity have been documented for Nipah virus [65,66], and reinfection-dependent severity gradients in Japanese encephalitis and dengue [67,68] are relevant given that W4–W5 patients may include individuals with prior SARS-CoV-2 exposure. The recurrence of this pattern across phylogenetically diverse viruses suggests that the divergence observed in our data reflects a conserved host response to neurotropic infection rather than a phenomenon unique to SARS-CoV-2.

Experimental models reinforce the biological plausibility of these observations. K18-hACE2 mice exhibit direct neuroinvasion with neuronal loss and microglial activation, higher viral loads correlating with greater thalamic and brainstem involvement, consistent with dose-dependent neuroinvasion [69,70]. Syrian hamsters demonstrate olfactory entry with progression to deeper CNS structures [14], offering a potential biological framework for the NE2-to-NE3 phenotypic shift. Non-human primates develop neuroinflammation, microhemorrhages, and α-synuclein aggregation [13,15], while neurotropism independent of respiratory severity was confirmed across additional mammalian hosts, including mink [11], cats, and ferrets [71,72]. At the molecular level, SARS-CoV-2 infects endothelial cells [39] and causes ACE2-dependent cerebral vascular dysfunction [73], aligning with the cerebrovascular enrichment observed in NE3. Although causal inference cannot be drawn from observational data, the convergence of clinical, epidemiological, and experimental evidence supports cerebrovascular injury as a plausible substrate for the progressive neurological trajectory documented in this cohort.

### 4.2. Systematic Confound Elimination and Stability of the Core Divergence

The stability of the NE3–PSY divergence was tested against eleven potential confounders (Table 5; Tables S7–S10). Sex, daylight exposure, and O_2_ mediation showed null effects, while pre-existing neurological comorbidity attenuated the NE3 wave effect by only 23.2%, leaving the majority unexplained by measured covariates. Importantly, clinical respiratory severity was inversely associated with NE3 (Table 5). This refutes a purely hypoxia-mediated injury model [74,75] and echoes respiratory–neurological dissociations documented in influenza-associated encephalopathy [47], MERS-CoV [76], and Nipah virus. Age-stratified analyses confirmed that the NE3 increasing trend was statistically significant within each demographic stratum where adequate power existed (ages 45–64, ≥65, <61, and ≥61) and directionally consistent in the youngest group (<45 years) despite limited sample size (Table S31). The PSY decline was partially explained by ICU-associated delirium [43,44] and corticosteroid-induced psychiatric effects [77,78] that diminished as protocols evolved following the RECOVERY Trial [20]. Nevertheless, we acknowledge that residual confounding by age and unmeasured age-related variables cannot be fully excluded in an observational design.

The inverse respiratory–neurological association has several potential explanations. Patients with severe respiratory disease may die before developing neurological complications, aggressive respiratory management may mask neurological symptoms, or NE3 may represent a distinct vulnerability profile rather than a severity-driven complication. The enrichment analysis, showing that NE3 patients were depleted for respiratory-associated codes and enriched for cerebrovascular pathology, supports this interpretation.

A related methodological consideration is detection quality. Neurological-psychiatric documentation concordance was low overall but improved progressively from early to later waves, and only a small fraction of patients with neurological symptoms received neurology consultation, possibly reflecting the limited availability of specialists during peak pandemic periods. The divergent documentation patterns between neurological and psychiatric phenotypes, with NE models performing better on symptom-level data and PSY models on clinical records, suggest that structured multi-source assessment protocols could improve recognition of both categories.

### 4.3. The Modified Neurological Severity Index (mNSI)—Validation and Clinical Utility

Beyond confounder robustness, the clinical coherence of the NE3 trajectory is further supported by a novel composite metric. The mNSI (integrating clinical neurological evolution and specialty-derived diagnostic confirmation) correlated strongly with mortality and ICU admission, adding independent predictive value beyond established risk markers, and increasing fivefold from early to later waves (Figure 4 and Figure 7; Table S11). This composite index may therefore capture the cumulative endothelial and neuroinflammatory burden that drives severe neurological outcomes beyond traditional respiratory markers. To our knowledge, few prior instruments have integrated multi-source data into a single continuous metric with demonstrated incremental mortality prediction [74,75]. Integrated classification models achieved discriminative performance ranging from good to excellent across phenotypes (Figure 7; Table S11).

### 4.4. ICD-10 Enrichment Signatures—Biological Validation of Cluster Separation

While the mNSI quantifies neurological severity as a continuous metric, the ICD-10 enrichment analysis provides complementary validation at the diagnostic-code level. NE3 showed significance in all 30 tested codes, with a cerebrovascular-dominant profile mirroring stroke-heavy descriptions [58,59,79]. The PSY cluster exhibited a clean F-chapter signature without G-chapter enrichment, supporting biological separation between psychiatric and neurological phenotypes and explaining discrepancies with broader EHR-based studies such as Taquet et al. [42], where comorbid patients contribute simultaneously to both categories. The emergence of toxic liver disease (K71.0) in the PSY cluster likely reflects hepatotoxicity from psychotropic medications [80], although direct causality could not be established in the present dataset. Analogous virus-specific neurological fingerprints are observed across flaviviruses [67,81,82], and the large NE3 effect sizes with simultaneous depletion of common COVID-associated codes establish NE3 as a discrete clinical entity with implications for registry design. This cerebrovascular-dominant signature aligns with endothelial infection and microvascular dysfunction [39,73] associated with the rise in NE3 independent of respiratory severity.

### 4.5. Cross-Coronavirus Neurological Legacy

Section 4.1.3 established that the NE3–PSY divergence has parallels across phylogenetically diverse neurotropic viruses. The closest phylogenetic relatives of SARS-CoV-2, however, merit separate examination because they share both receptor biology and beta coronavirus-specific neuroinvasion pathways. During the 2003 SARS outbreak, acute neurological involvement included polyneuropathy, myopathy, and ischemic stroke resembling NE3’s profile [83,84]. Psychiatric sequelae (PTSD 25.6%, depression 15.6%) were prominent early but gradually declined as neurological symptoms persisted [85]. Neuroinvasion occurred via the olfactory bulb with trans-synaptic spread [16,86], the same conserved route documented for SARS-CoV-2 [14]. MERS-CoV demonstrated that beta coronavirus neurotropism extends beyond the SARS lineage. Neurological complications affected 25.7% of critically ill patients [87], with confirmed CSF neuroinvasion [17]. Crucially, neurological events have been documented independently of significant respiratory involvement [76], while larger cohort analyses indicate neurological complications in a substantial proportion of MERS patients [18,87], paralleling our inverse CL_RE–NE3 association.

The receptor difference between ACE2 (SARS lineage) and DPP4 (MERS) may contribute to differences in predominant neuropathological patterns: DPP4 expression on neurons [88] favors encephalitis, while ACE2’s predominance on brain endothelial cells [39,73] is consistent with the cerebrovascular pathology defining NE3. Long-term SARS follow-up [89] further suggests that the acute NE3 trajectory may represent an early phase of longer-term neuropsychiatric burden.

### 4.6. Three-Phase Mechanistic Model

The preceding sections have established the NE3–PSY divergence (Section 4.1), its robustness to confounders (Section 4.2), its quantification via the mNSI (Section 4.3), its biological validation through ICD-10 signatures (Section 4.4), and its conservation across beta coronaviruses (Section 4.5). These convergent lines of evidence can be integrated into a three-phase mechanistic model mapping the NE3 rise and PSY decline onto distinct clinical drivers across the pandemic timeline.

In Phase I (W1–W2, 2020–2021), high initial PSY prevalence was driven by pandemic novelty, hospitalization anxiety, social isolation, cytokine-mediated delirium [43,90], and corticosteroid-induced psychiatric effects before RECOVERY-guided dosing. NE3 was rare, possibly because the ancestral strain had lower cerebrovascular tropism.

Phase II (W3–W4, 2021–2023) marks the transition: pandemic normalization did not reduce reactive psychiatric drivers in this phase. PSY remained relatively stable despite the overall downward trajectory across the full study period (Table 4). In contrast, NE3 rose sharply, potentially associated with greater variant-specific neurotropism [55] and cumulative CNS vulnerability from prior infections [54]. Diagnostic concordance also improved during this period, but pre-existing neurological comorbidity accounted for only a minority of the NE3 increase.

In Phase III (W5, 2023–2025), reactive psychiatric drivers had largely attenuated while NE3 reached its peak prevalence, likely driven by accumulated endothelial injury from prior infections and the older age profile of W5 patients. The progressive endothelial accumulation across reinfections thus offers a plausible biological substrate for the rising NE3 burden observed in our aging late-wave cohort.

The older age profile of W4–W5 patients (Table 1) reflects the evolving epidemiological context of the pandemic rather than a sampling artefact. During Omicron-dominant waves, milder disease in younger and vaccinated individuals reduced hospitalization rates in these groups, concentrating hospital admissions among older, often unvaccinated patients with accumulated comorbidities [91,92,93]. Romania’s national vaccination coverage plateaued at approximately 42% by mid-2022, with substantially lower uptake among the elderly rural population [94,95,96]. Consequently, W4–W5 hospital cohorts are enriched for older, potentially unvaccinated individuals, a pattern observed across multiple large cohort studies in Europe and the United States during the Omicron transition [6,97]. This demographic shift is a real feature of the later pandemic waves rather than a bias introduced by the study.

This model conceptually describes the relative visibility of concurrent processes as contextual psychiatric noise attenuates, revealing the underlying neurological signal, and predicts that future respiratory pandemics will exhibit a similar reactive-to-organic temporal sequence. Over successive waves, cumulative endothelial injury from repeated viral exposures thus appears to unmask a progressive neurological trajectory that outlasts the transient psychiatric component. The high-performing mortality model (Table S11) reinforces this interpretation: later waves carried a protective association with mortality despite rising NE3, a paradox that may be partially explained by improving clinical management [98].

#### Limitations

Several limitations warrant acknowledgment. (1) The single-center design limits generalizability, though this hospital served as the principal COVID-19 referral center for the county across all five waves. (2) The retrospective observational design precludes causal inference, particularly regarding corticosteroid–psychiatric and variant–neurological associations. (3) Wave-stratified random sampling from ~5000 hospitalizations may introduce selection bias. Inclusion criteria were applied uniformly, but W1 patients were distributed across multiple facilities according to evolving triage protocols, while W2–W5 concentrated at the study center, potentially enriching later waves for greater clinical severity and older age. (4) NLP-based extraction from Romanian-language electronic records, validated by manual review and cross-source concordance, may contain residual misclassification of nuanced neuropsychiatric presentations. (5) Due to the absence of direct virological data (variant sequencing, CSF viral loads), variant-related interpretations remain hypothesis-generating. (6) Wave × season confounding (χ^2^ = 404.7) cannot be fully disentangled, despite daylight analysis arguing against a major seasonal effect. (7) Vaccination status was available for 706/1424 patients (49.6%). Non-random missingness concentrated in W1–W2 with wave-adjusted analyses (N = 658) showing no independent vaccination–outcome association (all adj *p* > 0.10; Table S32), but incomplete coverage warrants cautious interpretation. (8) Despite significant cohort aging (mean 59.4 → 70.9 years), multivariable and age-stratified analyses suggest the NE3 trend is not solely demographic (Table S31); however, residual age confounding cannot be excluded. (9) The minimum detectable effect (Cramér’s V = 0.090 at 80% power) is well below the observed primary effect, but specific age × wave subgroups may be underpowered. (10) The mNSI requires prospective external validation before clinical implementation. (11) Pre-existing neurological comorbidity accounts for ~23% of the NE3 increase, requiring finer comorbidity characterization to clarify residual variance. (12) Immunological naivety could not be ascertained: participants hospitalized in later waves may have experienced prior unrecorded SARS-CoV-2 infections, and the resulting partial immunity could differentially modulate neuropsychiatric cluster profiles and trajectory patterns; the absence of a reliable infection-history registry precludes adjustment for this confounder. (13) Due to the retrospective design and reliance on hospital records, this cross-sectional hospitalization study captures neuropsychiatric manifestations at admission without post-discharge follow-up. Given that a substantial proportion of post-COVID neurological and psychiatric sequelae develop or evolve after the acute episode, particularly following mild infections in non-hospitalized patients, who constitute the majority of SARS-CoV-2 cases, the present findings may underestimate the full neuropsychiatric burden and cannot address long-term trajectories or chronic outcomes.

## 5. Conclusions

This five-wave, longitudinal study of 1471 hospitalized COVID-19 patients identifies a temporal divergence between declining psychiatric manifestations and increasing severe neurological phenotypes (NE3) across successive pandemic waves. Critically, this divergence was compositional: the aggregate neuropsychiatric burden remained stable while its internal composition shifted decisively from psychiatric to neurological dominance, a pattern not detectable in cross-sectional designs. The NE3 signal persisted after multivariable adjustment and within age-stratified subgroups, was inversely associated with respiratory severity (arguing against a purely hypoxia-mediated mechanism), and could not be attributed to vaccination status, although residual age confounding cannot be excluded. ICD-10 enrichment confirmed biological separation between clusters, with NE3 defined by a cerebrovascular-dominant signature distinct from typical COVID-19 presentations. A composite neurological severity index (mNSI) independently predicted mortality and ICU admission beyond established risk markers. The recurrence of analogous neurological–psychiatric dissociations across beta coronaviruses and phylogenetically diverse neurotropic infections suggests that this temporal pattern may reflect a conserved host response rather than a phenomenon unique to SARS-CoV-2. We propose a three-phase model—reactive psychiatric dominance, transitional crossover, and progressive neurological injury—that may generalize to future respiratory pandemics. These findings underscore the need for structured neuropsychiatric surveillance, prospective age-controlled studies, and external validation of composite severity indices to refine neurological risk stratification in emerging infectious diseases.

## Figures and Tables

**Table 1:** Neuropsychiatric cluster definitions.

Cluster	Label	N	Weight	Clinical Definition
**NE2**	Moderate neurological	12	7	Headache, vertigo, dizziness, anosmia, ageusia, paresthesia, tremor, somnolence, dysarthria, amnesia
**NE3**	Severe neurological	7	9–10	Confusion, seizures, paresis, aphasia, hemiparesis, motor deficit, obtundation
**PSY1**	Mild psychiatric	5	4	Anxiety, insomnia, depression
**PSY2**	Moderate psychiatric	2	5	Psychomotor agitation, panic attacks
**CA**	Cardiovascular	7	7–9	Palpitations, chest pain, edema, arrhythmia
**RE**	Respiratory	9	4–10	Cough, dyspnea, desaturation, wheezing
**NC**	Non-classified	40	0	Constitutional, GI, urinary, dermatological, hepatic
**NECA**	Neuro-cardiovascular	2	9	Syncope (dual neuro/cardio classification)
**NEPSY3**	Neuro-psychiatric	2	8	Hallucinations (dual neuro/psychiatric classification)

**Table 2:** Cluster-level International Classification of Diseases, 10th Revision (ICD-10) enrichment signatures.

Cluster	Sig/30	Type	Top Enrichment (OR)	Key ICD Chapters
NE2 (moderate neuro)	19/30	Symptom	R51 Headache (19.6)	Symptoms/Signs (R), Circ (I)
NE3 (severe neuro)	30/30 **	Symptom	R47.0 Dysphasia (182.8)	Nervous Sys (G), Circ (I)
NEPSY3	1/30	Symptom	Z72.1 (48.3)	—
PSY (psychiatric)	10/30	Symptom	F41.2 Mixed anxiety (10.1)	Mental/Behavioral (F)
NEURO (any neuro)	9/30	Symptom	R51 Headache (14.9)	Symptoms/Signs (R)
NEUROPSY (any NP)	17/30	Symptom	R51 Headache (14.9)	Symptoms/Signs (R), G
SPEC_NE	30/30 **	Clinical	G31.0 Neurodegen (18.6)	Nervous Sys (G), Circ (I)
SPEC_PSY	2/30	Clinical	G31.0 (5.3), G46.7 (9.1)	Nervous System (G)
ANY_NE	26/30	Clinical	G31.0 (26.7)	Nervous Sys (G), Circ (I)
ANY_PSY	18/30	Clinical	F43.0 Acute stress (31.7)	Mental/Behavioral (F), G
ANY_NEUROPSY	15/30	Clinical	G31.0 (17.5)	Nervous System (G)
COMBINED_NE	12/30	Combined	R51 Headache (12.5)	Symptoms/Signs (R), G
COMBINED_PSY	18/30	Combined	F43.0 Acute stress (34.7)	Mental/Behavioral (F), G
COMBINED_NEUROPSY	11/30	Combined	R51 Headache (11.6)	Symptoms/Signs (R), G

** maximum significance—30 out of 30 tested ICD-10 codes reached statistical significance. Secondary diagnosis enrichment analysis was consistent with distinct biological coding profiles across clusters: NE3 patients’ ICD-10 comorbidity was dominated by cerebrovascular and motor-deficit codes, while showing relative depletion of typical COVID accompaniments. PSY patients showed a predominantly F-chapter (mental/behavioral) profile with no neurological code enrichments, supporting complete biological separation between neurological and psychiatric phenotypes at the secondary diagnosis level (Table 2).

**Table 3:** Demographic, Clinical, and Neuropsychiatric (NeuroPsy) Characteristics by Pandemic Wave (N = 1471).

Characteristic	Total (N = 1471)	W1: Wild (n = 345)	W2: Alpha (n = 247)	W3: Delta (n = 274)	W4: OmEarly (n = 282)	W5: OmLate (n = 323)	*p*
Demographics							
Age, mean (SD)	63.6 (15.6)	59.4 (14.0)	58.6 (14.7)	59.6 (14.9)	68.9 (16.8)	70.9 (13.2)	<0.001 ***
Age, median [IQR]	66 [53–75]	61 [50–69]	60 [49–69]	62 [50–70]	72 [61–82]	73 [66–79]	<0.001 ***
Male sex, n (%)	668 (45.4%)	147 (42.6%)	113 (45.7%)	119 (43.4%)	129 (45.7%)	160 (49.5%)	0.44
Hosp. days, mean (SD)	11.8 (6.8)	15.6 (6.8)	12.4 (6.0)	13.6 (6.6)	8.7 (4.8)	8.3 (6.3)	<0.001 ***
Hosp. days, median [IQR]	11 [7–14]	14 [11–19]	11 [9–14]	13 [10–15]	7 [6–10]	6 [5–9]	<0.001 ***
Secondary dx, mean (SD)	14.2 (5.3)	13.9 (4.4)	15.3 (4.7)	15.8 (5.2)	14.1 (5.1)	12.5 (6.2)	<0.001 ***
Severity							
ICU admission, n (%)	194 (13.2%)	41 (11.9%)	31 (12.6%)	39 (14.2%)	41 (14.5%)	42 (13.0%)	0.86
Any O_2_ support, n (%)	476 (32.4%)	77 (22.3%)	103 (41.7%)	133 (48.5%)	83 (29.4%)	80 (24.8%)	<0.001 ***
In-hospital mortality, n (%)	131 (8.9%)	35 (10.1%)	22 (8.9%)	24 (8.8%)	22 (7.8%)	28 (8.7%)	0.89
NeuroPsy outcomes (Symptom tier)							
Severe neurological (NE3)	133 (9.0%)	12 (3.5%)	7 (2.8%)	8 (2.9%)	41 (14.5%)	65 (20.1%)	<0.001 ***
Mild psychiatric (PSY1)	129 (8.8%)	46 (13.3%)	27 (10.9%)	19 (6.9%)	23 (8.2%)	14 (4.3%)	<0.001 ***
Mild-mod neurological (NE2)	663 (45.1%)	151 (43.8%)	124 (50.2%)	160 (58.4%)	116 (41.1%)	112 (34.7%)	<0.001 ***
Moderate psychiatric (PSY2)	43 (2.9%)	13 (3.8%)	8 (3.2%)	9 (3.3%)	4 (1.4%)	9 (2.8%)	0.50
Any neurological (grouped)	743 (50.5%)	162 (46.9%)	131 (53.0%)	163 (59.5%)	137 (48.6%)	150 (46.4%)	0.008 **
Any psychiatric (grouped)	163 (11.1%)	58 (16.8%)	33 (13.4%)	24 (8.8%)	27 (9.6%)	21 (6.5%)	<0.001 ***
Any neuropsychiatric (grouped)	809 (55.0%)	187 (54.2%)	148 (59.9%)	171 (62.4%)	146 (51.8%)	157 (48.6%)	0.004 **
NeuroPsy outcomes (Clinical tier)							
Any clinical neurological	401 (27.3%)	59 (17.1%)	72 (29.1%)	59 (21.5%)	99 (35.1%)	112 (34.7%)	<0.001 ***
Any clinical psychiatric	237 (16.1%)	84 (24.3%)	30 (12.1%)	35 (12.8%)	35 (12.4%)	53 (16.4%)	<0.001 ***
Any clinical neuropsychiatric	521 (35.4%)	114 (33.0%)	88 (35.6%)	83 (30.3%)	107 (37.9%)	129 (39.9%)	0.05
Neurology consultation	150 (10.2%)	13 (3.8%)	12 (4.9%)	12 (4.4%)	59 (20.9%)	54 (16.7%)	<0.001 ***
Psychiatry consultation	62 (4.2%)	22 (6.4%)	13 (5.3%)	11 (4.0%)	7 (2.5%)	9 (2.8%)	—

Continuous variables: Kruskal–Wallis H test. Categorical variables: Pearson χ^2^ test with BH-adjusted *p*-values. ** *p* < 0.01, *** *p* < 0.001.

**Table 4:** Core neuropsychiatric prevalence and adjusted ORs by pandemic wave (N = 1471).

Variable	Tier	W1 (%)	W2 (%)	W3 (%)	W4 (%)	W5 (%)	χ^2^ (4)	*p*	V	W5 Adj OR	Adj *p*
NE3	S	3.5	2.8	2.9	14.5	20.1	95.6	8.3 × 10^−20^	0.255	4.34	1.4 × 10^−4^
PSY1	S	13.3	10.9	6.9	8.2	4.3	19.7	5.9 × 10^−4^	0.116	0.16	2.2 × 10^−8^
ANY_NEURO	S	47.0	53.0	59.5	48.6	46.4	13.8	0.008	0.097	1.37 ^b^	0.194
ANY_PSY	S	16.8	13.4	8.8	9.6	6.5	21.8	0.0002	0.122	0.18	1.1 × 10^−8^
SPEC_NE	C	5.8	4.9	5.5	24.1	18.6	88.6	2.6 × 10^−18^	0.245	1.35 ^c^	0.383
SPEC_PSY	C	7.0	6.1	4.7	6.4	5.6	1.5	0.825	0.032	—	—
cross_NE_both	CS	10.1	17.0	15.3	21.3	24.8	28.3	1.1 × 10^−5^	0.139	—	—
cross_PSY_both	CS	10.4	3.6	5.8	2.5	4.6	22.7	1.5 × 10^−4^	0.124	—	—

Adj OR: logistic regression adjusting for age, hospitalization days, secondary diagnoses, ICU, O_2_, complication count, resolution (N = 708); reference = Wave 1. ^b^ ANY_NEURO W5 not individually significant, but overall wave block highly significant (Δχ^2^ = 35.9, *p* = 3.0 × 10^−7^); dominant effect at W3 (adj OR = 0.26, *p* < 0.001). ^c^ SPEC_NE W5 no longer significant after convergence correction; W4 remains significant (adj OR = 5.46, *p* = 0.003). S—symptom; C—clinical; CS—cross-source.

**Table 5:** Systematic confound elimination for the NE3 wave effect (N = 1471).

Confound	Analysis	Effect on NE3	*p*	Verdict
Sex	Main effect + interactions	OR = 1.285	0.21	Null for NE3 (sig for PSY)
Season	χ^2^ incidence	*p* = 0.034	0.034	Sig but confounded (χ^2^ = 404.7)
Daylight	Logistic; ORs	~0.95	>0.05	Null (3/18 borderline)
O_2_ support	Mediation (path a)	*p* = 0.44	0.44	Null (no mediator var.)
CL_RE respiratory	Logistic	OR = 0.704	0.0001	Inverse (resp ↑ → NE3 ↓) *
Neuro-comorbidity	Attenuation	23.2% attenuated	—	Partial—76.8% persists
Neurodegenerative dx	Attenuation	−0.1%	—	Not explanatory
ICD-10 chapter	χ^2^; logistic	J vs. non-J: OR = 0.608	0.041	Non-J → higher NE3
Wave × sex	LR interaction	—	0.023	1/44 sig (SPEC_NE only)
Wave × age_group	LR interaction	—	2 × 10^−6^	2/44 sig (ANY_NE, NEUROPSY)
Wave × high_resp	LR interaction	—	0.003	2/44 sig (PSY, SPEC_HAS_NEU)

* Resp ↑—respiratory severity increase inversely correlated with NE 3 (↓— severe neurological clinical symptom cluster decrease).

**Table 6:** Classification performance of integrated models.

Model	AUC	Pseudo-R^2^	Cross-Val AUC (±SD)	Dominant Block (ΔR^2^)
has2_ANY_NE	0.761	0.309	0.733 ± 0.036	Specialty (0.230)
has2_ANY_PSY	0.841	0.375	0.763 ± 0.016	Behavioral (0.120)
has2_ANY_NEUROPSY	0.788	0.329	0.710 ± 0.062	—
Mortality (EVO_DEATH)	0.918	0.494	—	ICU + O_2_

## Data Availability

The data presented in this study are available from the corresponding author upon reasonable request. The data is not publicly available due to patient privacy and institutional regulations.

## Supplementary Materials

The following supporting information can be downloaded at: https://www.mdpi.com/article/10.3390/jcm15083030/s1. S1: Medications Dictionary, S2: Physical Exams Dictionary, S3: Symptoms Dictionary, Figure S1: Symptom Heatmap; Figure S2: Tier 1 Cluster Prevalence by Wave Barplot, Figure S3: Tier 2 Clinical-Level Prevalence by Wave Barplot, Figure S4: Tier 3 Cross-Source Agreement Prevalence by Wave Barplot, Figure S5: Unadjusted vs. Adjusted Symptom Cluster Odds Ratios, Figure S6: Wave Odds Ratios Across Model Specifications, Figure S7: Prevalence by Subgroup and Wave, Figure S8: Daylight Median Odds Ratio across models, Figure S9: Daylight Tercile Distribution by Pandemic Wave, Figure S10: Respiratory, Cardiac and Neuropsychiatric Measures Correlation, Figure S11: Oxygen Support Levels by Pandemic Wave, Figure S12: Daylight Median vs. Neuropsychiatric Weights, Figure S13: Cerebral Burden vs. Number of Specialty Consults, Figure S14: Neurological Comorbidity Burden by Wave, Figure S15: Predicted P by O_2_ level and Corticosteroid Correlation, Figure S16: Discharge Medication Profile by Neuropsychological Status, Figure S17: Daylight Median by Neuropsychiatric Outcome Status, Figure S18: Daylight and Age interaction on Neurological Specialty Consult Cluster 2 Weight, Table S1: Tier 1 Cluster Incidence, Table S2: Tiered Clusters Odds Ratios, Table S3: Tier 2 Clinical Indicator Incidence, Table S4: Tier 3 Cross-Source Clinical Indicator Incidence, Table S5: Multivariate Models, Table S6: All Coefficients Multivariate Processing, Table S7: Variance Inflation Factors per multiple coefficients, Table S8: Wave DV per Odds Ratios, Table S9: Neuropsy Interactions with age, Sex, Evolution and Respiratory Support, Table S10: Respiratory Predictors, Table S11: O_2_ Mediation on Clinical Neuropsy, Table S12: Statistical Methods Checklist, Table S13: Phenotype Chi Square, Table S14: Discharge Medication to Neuropsy Associations, Table S15: Corticosteroid to O_2_ Associations, Table S16: Neuropsy to predictors cross-validation, Table S17: Mortality Model, Table S18: Backward Elimination NE Validation, Table S19: Multiple Model Predictor Assessments, Table S20: Cluster Chapter Distribution, Table S21: Concordance Predictors x Neuropsy, Table S22: Seasonality and Daylight Cluster Associations, Table S23: Neuropsy Multiple Coefficients Interactions by Wave, Table S24: Neuropsy Clinical and Specialty Consult ICD J code prevalence, Table S25: Neuropsy Clinical and Specialty Consult ICD G, F Code Interactions, Table S26: Tier 1 Grouped Neuropsy Incidence, Table S27: Sex effect summary on Neuropsy, Table S28: Neuropsy Severity trends by O_2_, ICU, Stroke and Behaviour, Table S29: O_2_ Stratified Incidence by Cluster Indicators, Table S30: Significant Enrichments by Clusters, Table S31: Age Stratified NE3 and PSY trends, Table S32: Vaccination Outcomes, Severity, CMH and NE3 logistic regression model.

## Abbreviations

The following abbreviations are used in this manuscript:

ACE2	Angiotensin-converting enzyme 2
AEM	Anti-epileptic medication
AUC	Area under the curve
BH	Benjamin-Hochberg correction
cART	Combination antiretroviral therapy
CL_NE	Clinical Neurological cluster score
CL_PSY	Clinical Psychiatric cluster score
CL_RE	Clinical Respiratory cluster score
CNS	Central nervous system
CNSI	Composite Neuropsychiatric Severity Index
CSF	Cerebrospinal fluid
DISC_	Discharge-documented findings
DISC_PSY	Discharge-documented psychiatric findings
DDP4	Dipeptidyl peptidase 4
EHR	Electronic health records
EVO_	Evolution-documented findings
EVO_PSY	Evolution-documented psychiatric findings
ICD-10	International classification of Diseases, 10th edition
ICU	Intensive care unit
LR	Likelihood ratio
mNSI	Modified Neurological Severity Index
NCB	Neurological comorbidity burden
NE2	Moderate neurological cluster
NE3	Severe neurological cluster
NECA	Neuro-cardiac cluster
NEPSY3	Combined neuropsychiatric severe cluster
NLP	Natural language processing
OR	Odds ratio
PSY1	Mild psychiatric cluster
PSY2	Moderate psychiatric cluster
RAT	Rapid antigen test
RECOVERY	Randomized Evaluation of COVID-19 Therapy–trial
SPEC_	Specialty consultation documentation
SPEC_NE	Specialty neurological consultation indicator
VIF	Variance inflation factor
