# Supplementary material for: Psychiatric and Neurological Involvement in COVID-19 Hospitalized Patients Through the Global Pandemic in Central Romania
Source: J Clin Med. 2026 Apr 16;15(8):3030. doi: 10.3390/jcm15083030 (PMC13116866; doi:10.3390/jcm15083030)
Supplement: Supplementary file 1 [file jcm-15-03030-s001.zip › Table_S12_Statistical_Tests_Reference.html]

Statistical Tests Reference — Article 1

# Statistical Tests & Diagnostic Metrics Reference

Article 1 — Psychiatric and Neurological Involvement in COVID-19 • N = 1,471 • 43 methods (22 tests + 21 metrics)

43

Total Methods

22

Statistical Tests

21

Diagnostic Metrics

17

Hypothesis Tests

7

Effect Sizes

6

Model Validation

Hypothesis (17)

Effect (7)

Model (4)

Multiple (1)

Model (6)

Exploratory (6)

Descriptive (2)

Filter by Type:

All (43)
Statistical Tests (22)
Diagnostic Metrics (21)

Filter by Category:

All (43)
Hypothesis Tests (17)Effect Sizes (7)Model Diagnostics (4)Multiple Comparisons (1)Model Validation (6)Exploratory Methods (6)Descriptive Statistics (2)

| # | Test / Method | Category | Type | Where Used | Rationale | What Was Analyzed |
| --- | --- | --- | --- | --- | --- | --- |
| 1 | **Pearson chi-squared test** | Hypothesis Tests | Statistical Test | Table 3, Table 4, Section 3.1.1, Section 3.2.2, Section 3.5.1-2; S1, S3, S4, S14, S24, S29, S31, S33 | *The five-wave structure and multi-category diagnoses produced large contingency tables with adequate cell counts, suited for chi-squared testing of categorical associations.* | Cross-tabulated neuropsychiatric diagnoses by pandemic wave, sex, and clinical subgroups. |
| 2 | **Fisher exact test** | Hypothesis Tests | Statistical Test | Post-hoc wave pairs, small cells; S2, S27, S33 | *Pairwise wave comparisons and rare diagnosis subcategories produced cells with fewer than 5 expected counts, necessitating exact inference.* | Pairwise wave comparisons and rare diagnosis categories with sparse cell counts. |
| 3 | **Cochran-Armitage trend test** | Hypothesis Tests | Statistical Test | Section 3.1.1 (Z = 8.68); S1, S3, S4, S29, S32, S34 | *Pandemic waves form a natural ordinal sequence; this test was needed to detect monotonic trends in neuropsychiatric incidence across the five waves.* | Linear trends in neuropsychiatric incidence rates across the five pandemic waves. |
| 4 | **Kruskal-Wallis H test** | Hypothesis Tests | Statistical Test | Table 3, Section 3.4.1 mNSI across waves; S24, S31 | *mNSI scores and hospitalization days were non-normally distributed; comparing these across five waves required a non-parametric multi-group test.* | mNSI severity scores and hospitalization days compared across five pandemic waves. |
| 5 | **Mann-Whitney U test** | Hypothesis Tests | Statistical Test | Section 3.4.1 mNSI vs mortality/ICU; S10 | *mNSI severity scores were skewed; comparing two groups (survivors vs deceased, ICU vs non-ICU) required a non-parametric two-sample test.* | mNSI scores between survivors vs non-survivors and ICU vs non-ICU patients. |
| 6 | **McNemar test** | Hypothesis Tests | Statistical Test | S23 Section 2 (detection asymmetry) | *Symptom-level and clinical-level classification were applied to the same 1471 patients, creating paired data that required a test for systematic detection asymmetry.* | Detection asymmetry between symptom-level and clinical-level diagnostic classification. |
| 7 | **Mantel-Haenszel stratified OR** | Hypothesis Tests | Statistical Test | Section 3.2.5 vaccination (MH OR = 0.97) | *Vaccination rates varied sharply across waves; isolating the vaccination-neuropsychiatric association required stratification by wave to control confounding.* | Vaccination effect on neuropsychiatric outcomes stratified by pandemic wave. |
| 8 | **Binary logistic regression (ML)** | Hypothesis Tests | Statistical Test | Section 3.1.2, Table 4; S5, S6, S8, S10, S16, S18, S19, S30 | *Multiple binary outcomes (neuropsychiatric diagnosis, ICU admission, mortality) needed simultaneous adjustment for demographic, clinical, and wave-related predictors.* | Predictors of neuropsychiatric diagnosis, ICU admission, and in-hospital mortality. |
| 9 | **Hosmer-Lemeshow goodness-of-fit** | Hypothesis Tests | Diagnostic Metric | Section 3.1.2 (all p > 0.05); S5 | *With 1471 patients and multiple logistic models, formal calibration testing ensured that predicted risks aligned with observed event rates across deciles.* | Model fit for all multivariate logistic regression models (all p > 0.05 indicating adequate calibration). |
| 10 | **Likelihood-ratio test (nested)** | Hypothesis Tests | Statistical Test | Section 3.1.2 (delta-chi-squared = 39.2/51.8); S5, S9, S26, S28, S30 | *Wave-level and covariate-block models were built sequentially; LR tests determined whether each additional predictor block significantly improved model fit.* | Wave-level and covariate-block improvements in logistic regression model fit. |
| 11 | **Wald z-test (coefficient significance)** | Hypothesis Tests | Statistical Test | Implicit in all logistic models; S6 | *Each predictor's individual contribution needed assessment within the multivariate logistic framework to identify significant risk and protective factors.* | Statistical significance of each predictor in all multivariate models. |
| 12 | **Baron-Kenny mediation + bootstrap CI** | Hypothesis Tests | Statistical Test | Section 3.2.3 (bootstrap CI non-significant); S11 | *Oxygen support co-varied with both wave and neuropsychiatric outcomes; mediation analysis with percentile bootstrap (5,000 resamples) tested whether O₂ level explained the wave–outcome association.* | Whether supplemental oxygen mediated the wave–neuropsychiatric association (non-significant; all 95% CIs contain zero). |
| 13 | **OLS regression** | Hypothesis Tests | Statistical Test | Section 2.4.3 detection; S24 Section 4 (daylight) | *Detection validity and daylight-seasonality effects involved continuous outcomes requiring standard linear regression.* | Detection validity models and daylight-seasonality linear associations. |
| 14 | **Backward-elimination logistic regression** | Hypothesis Tests | Statistical Test | Section 3.6; S18, S19 | *Full models contained numerous predictors; automated backward elimination identified the most parsimonious predictor set for mortality and ICU prediction.* | Parsimonious mortality and ICU admission prediction models. |
| 15 | **Wave x covariate interaction tests** | Hypothesis Tests | Statistical Test | Section 3.2.7 (44 terms); S9, S26, S28 | *Risk-factor profiles were hypothesized to shift across pandemic waves; 44 interaction terms tested whether predictor effects were wave-dependent.* | 44 wave x covariate interaction terms testing differential neuropsychiatric risk patterns. |
| 16 | **Corticosteroid x O2 interaction** | Hypothesis Tests | Statistical Test | Section 3.5.2; S16 | *Corticosteroid protocols changed over the pandemic and their neuropsychiatric effects may differ by respiratory severity, requiring a targeted interaction test.* | Effect modification between corticosteroid use and supplemental oxygen on neuropsychiatric risk. |
| 17 | **Post-hoc power analysis** | Hypothesis Tests | Diagnostic Metric | Section 2.1 (>99% power for NE3) | *Reviewers expect power justification; post-hoc analysis confirmed >99% power for the primary neurological outcome given N = 1471.* | Power exceeding 99% for the primary neurological outcome (NE3) given N = 1471. |
| 18 | **Cramer V** | Effect Sizes | Diagnostic Metric | Table 4, Section 3.1.1, Section 3.1.3; S1, S3, S4, S29, S34 | *Chi-squared p-values alone do not convey clinical relevance; Cramer V quantified the practical strength of wave-diagnosis associations.* | Strength of association between pandemic wave and each neuropsychiatric diagnostic category. |
| 19 | **Odds ratio with 95% CI** | Effect Sizes | Diagnostic Metric | Throughout; all S tables with logistic models | *Binary logistic models yield ORs as the natural effect measure; CIs were essential for communicating the precision of each risk estimate.* | Risk estimates for all logistic regression predictors and bivariate comparisons. |
| 20 | **Cohen kappa** | Effect Sizes | Diagnostic Metric | Section 3.3.2 (kappa = 0.153/0.327); S23 Section 1 | *Two classification tiers (symptom-level vs clinical-level) were applied to the same patients; kappa quantified agreement beyond chance.* | Concordance between symptom-level and clinical-level neuropsychiatric detection. |
| 21 | **Kappa trajectory across waves** | Effect Sizes | Diagnostic Metric | Section 3.3.2 (W1 = 0.088 to W5 = 0.354) | *Diagnostic practices evolved as the pandemic progressed; tracking kappa across waves revealed whether detection agreement improved over time.* | Wave-specific kappa values from W1 (0.088) to W5 (0.354) showing improving concordance. |
| 22 | **Spearman rho** | Effect Sizes | Statistical Test | Section 3.7 (CNSI: rho = 0.43, 0.59); S24 Section 3 | *The composite severity index (CNSI) and clinical outcomes are ordinal/non-normal; Spearman rho assessed their monotonic relationship.* | Correlation between composite neuropsychiatric severity indices (CNSI) and clinical outcomes. |
| 23 | **Rank-biserial r** | Effect Sizes | Diagnostic Metric | S10 (MWU effect size) | *Mann-Whitney U tests need an accompanying effect size; rank-biserial r quantified the magnitude of respiratory-burden group differences.* | Magnitude of group differences in respiratory burden scores (Table S10). |
| 24 | **Percent attenuation** | Effect Sizes | Diagnostic Metric | Section 3.2.4 (23.2% attenuation) | *Hospitalization duration was a suspected confounder; percent attenuation measured how much of the crude wave-diagnosis OR it explained (23.2%).* | Confounding contribution of hospitalization duration to wave-diagnosis associations (23.2%). |
| 25 | **Variance Inflation Factor (VIF)** | Model Diagnostics | Diagnostic Metric | Section 3.1.2; S7 | *Age, secondary diagnoses, and hospitalization days were correlated; VIF checked whether multicollinearity threatened coefficient stability.* | Collinearity diagnostics for age, secondary diagnoses, and hospitalization days (VIF up to 13.82). |
| 26 | **McFadden pseudo-R-squared** | Model Diagnostics | Diagnostic Metric | S5, S6; Section 2.4.5 | *Logistic regression lacks a true R-squared; McFadden's approximation indicated overall explanatory power of each multivariate model.* | Overall explanatory power of each multivariate logistic model. |
| 27 | **AIC (Akaike Information Criterion)** | Model Diagnostics | Diagnostic Metric | S5, S6, S9, S26, S28 | *Multiple nested and interaction-augmented models required a parsimony criterion to select the best-fitting model without overfitting.* | Model comparison across nested and interaction-augmented logistic regressions. |
| 28 | **BIC (Bayesian Information Criterion)** | Model Diagnostics | Diagnostic Metric | S5, S6 | *With N = 1471, BIC's stronger complexity penalty complemented AIC for robust model selection.* | Model selection alongside AIC for robustness of model parsimony decisions. |
| 29 | **Benjamini-Hochberg FDR correction** | Multiple Comparisons | Diagnostic Metric | Throughout incidence; S1, S3, S4, S29, S32, S33, S34 | *Testing incidence trends across multiple diagnosis categories and waves created a multiplicity problem; FDR correction controlled false discoveries.* | Correction for multiple comparisons across wave x diagnosis incidence tests and enrichment analyses. |
| 30 | **5-fold stratified cross-validation** | Model Validation | Diagnostic Metric | Section 3.6; S17 | *Prediction models for mortality and ICU admission needed internal validation to estimate out-of-sample performance with balanced folds.* | Internal validation of mortality and ICU admission prediction models. |
| 31 | **AUC (area under ROC curve)** | Model Validation | Diagnostic Metric | Section 3.6; S17, S18, S19 | *Discrimination between fatal/non-fatal and ICU/non-ICU outcomes was the primary validation metric for the prediction models.* | Discriminative performance of mortality prediction models (AUC = 0.86 vs 0.76). |
| 32 | **Brier score** | Model Validation | Diagnostic Metric | S17 | *Beyond discrimination, calibration accuracy of predicted mortality probabilities was assessed via mean squared prediction error.* | Calibration quality of cross-validated mortality prediction models. |
| 33 | **Calibration slope** | Model Validation | Diagnostic Metric | S17 | *A calibration slope near 1.0 confirmed that predicted probabilities and observed event rates agreed, avoiding systematic over- or under-estimation.* | Agreement between predicted and observed mortality probabilities. |
| 34 | **Sensitivity / Specificity** | Model Validation | Diagnostic Metric | Section 3.3.2 (NE: 64.6%/54.8%; PSY: 35.0%/93.5%); S23 Section 5 | *The two-tier detection system required formal accuracy assessment to determine how well symptom-level screening identified confirmed diagnoses.* | Diagnostic accuracy of symptom-level vs clinical-level neuropsychiatric detection. |
| 35 | **PPV / NPV** | Model Validation | Diagnostic Metric | S23 Section 5 | *Given the cohort's disease prevalence, predictive values quantified the clinical utility of positive and negative detection-tier classifications.* | Predictive accuracy of detection tiers for confirmed neuropsychiatric diagnoses. |
| 36 | **PCA (principal component analysis)** | Exploratory Methods | Statistical Test | Section 3.7 (CNSI construction) | *Multiple correlated clinical indicators needed reduction into a single composite index (CNSI) that captured maximal variance for severity phenotyping.* | Construction of the Composite Neuropsychiatric Severity Index (CNSI) from clinical variables. |
| 37 | **Hierarchical clustering (Ward method)** | Exploratory Methods | Statistical Test | Section 3.7 | *After PCA-derived severity indices, Ward's clustering grouped patients into clinically distinct phenotype clusters for targeted analysis.* | Patient phenotype clustering based on neuropsychiatric severity profiles. |
| 38 | **Age-stratified subgroup analysis** | Exploratory Methods | Statistical Test | Section 3.1.1; S34 | *Neuropsychiatric susceptibility varies with age; stratification separated trends in young, middle-aged, and elderly subgroups.* | Neuropsychiatric incidence trends in young, middle-aged, and elderly subgroups. |
| 39 | **O2-stratified subgroup analysis** | Exploratory Methods | Statistical Test | S32 | *Supplemental oxygen proxies respiratory severity and may confound neuropsychiatric associations; stratification isolated its effect.* | Neuropsychiatric outcomes in patients with and without supplemental oxygen. |
| 40 | **ICU-stratified subgroup analysis** | Exploratory Methods | Statistical Test | S31 | *ICU patients represent a severity extreme; separate analysis ensured general-ward patterns were not driven by critically ill outliers.* | Neuropsychiatric outcomes in ICU vs non-ICU hospitalized patients. |
| 41 | **Sequential domain-block entry** | Exploratory Methods | Statistical Test | Section 3.6; S18, S19 | *Demographic, clinical, and treatment predictors were entered hierarchically to quantify each domain's incremental contribution to mortality prediction.* | Stepwise addition of demographic, clinical, and treatment blocks to mortality models. |
| 42 | **Mean / SD for continuous variables** | Descriptive Statistics | Diagnostic Metric | Table 3 | *Continuous variables (age, hospitalization days, severity scores) required summary statistics for cohort characterization in Table 3.* | Age, hospitalization days, and severity scores across the study cohort. |
| 43 | **Frequencies / percentages for categorical variables** | Descriptive Statistics | Diagnostic Metric | Table 3 | *Categorical variables (sex, wave, diagnoses, ICU, mortality) required counts and percentages for cohort description in Table 3.* | Sex, wave membership, diagnosis categories, ICU admission, and mortality rates. |
