# Supplementary figures and images for: Psychiatric and Neurological Involvement in COVID-19 Hospitalized Patients Through the Global Pandemic in Central Romania
Source: J Clin Med. 2026 Apr 16;15(8):3030. doi: 10.3390/jcm15083030 (PMC13116866; doi:10.3390/jcm15083030)

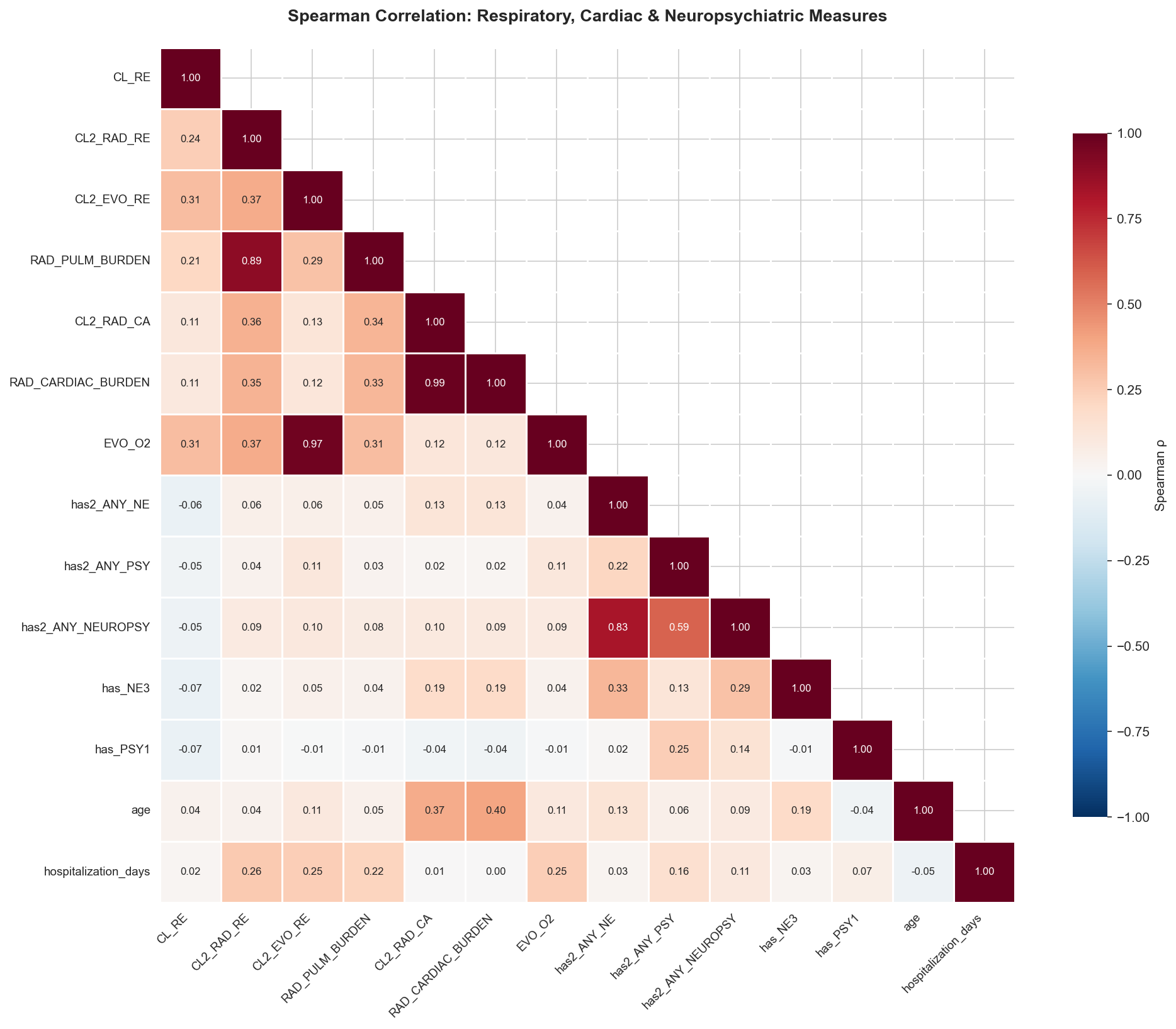

Supplement: Supplementary file 1 [file jcm-15-03030-s001.zip › Figure_S10_respiratory_correlation.png]

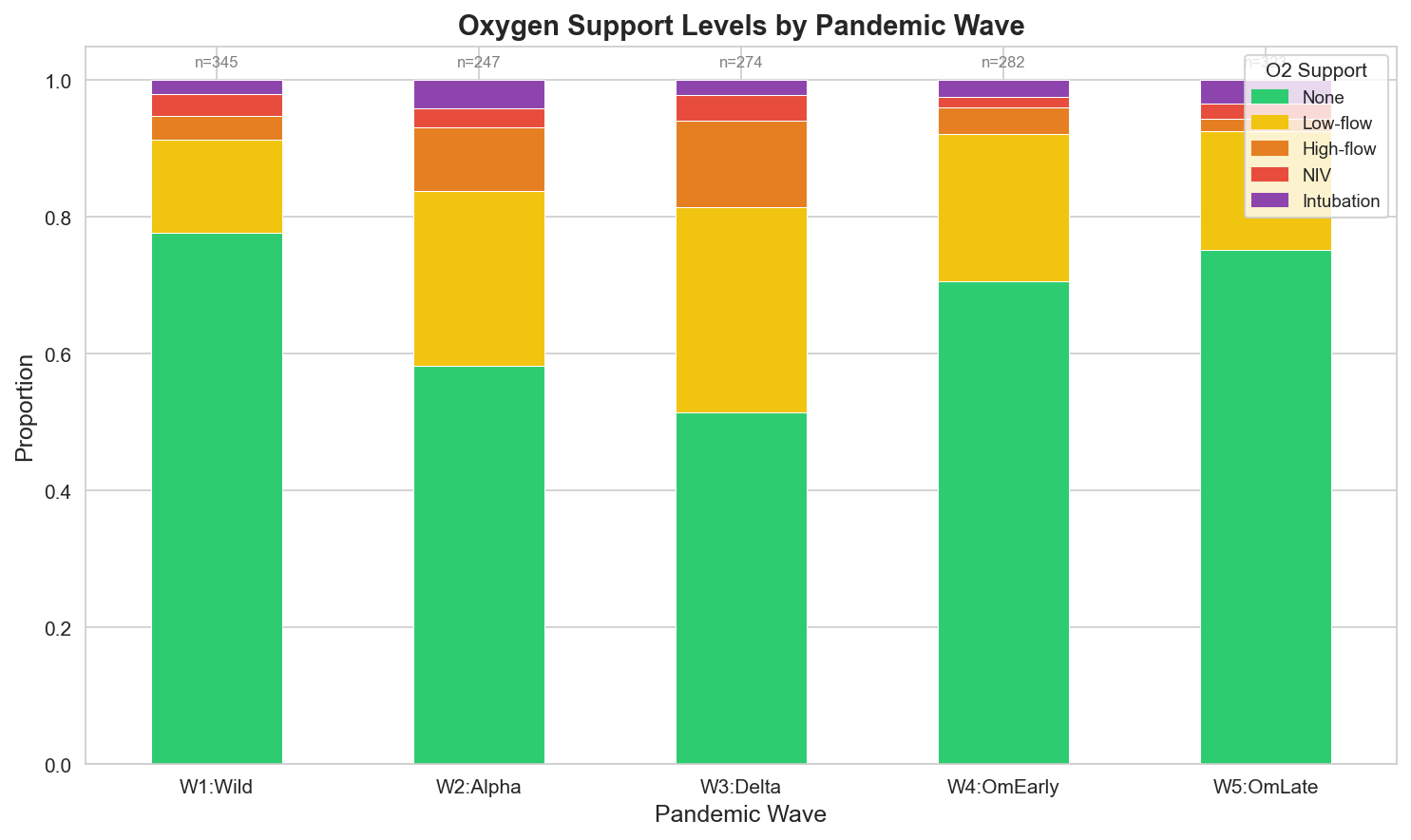

Supplement: Supplementary file 1 [file jcm-15-03030-s001.zip › Figure_S11_evo_o2_by_wave.png]

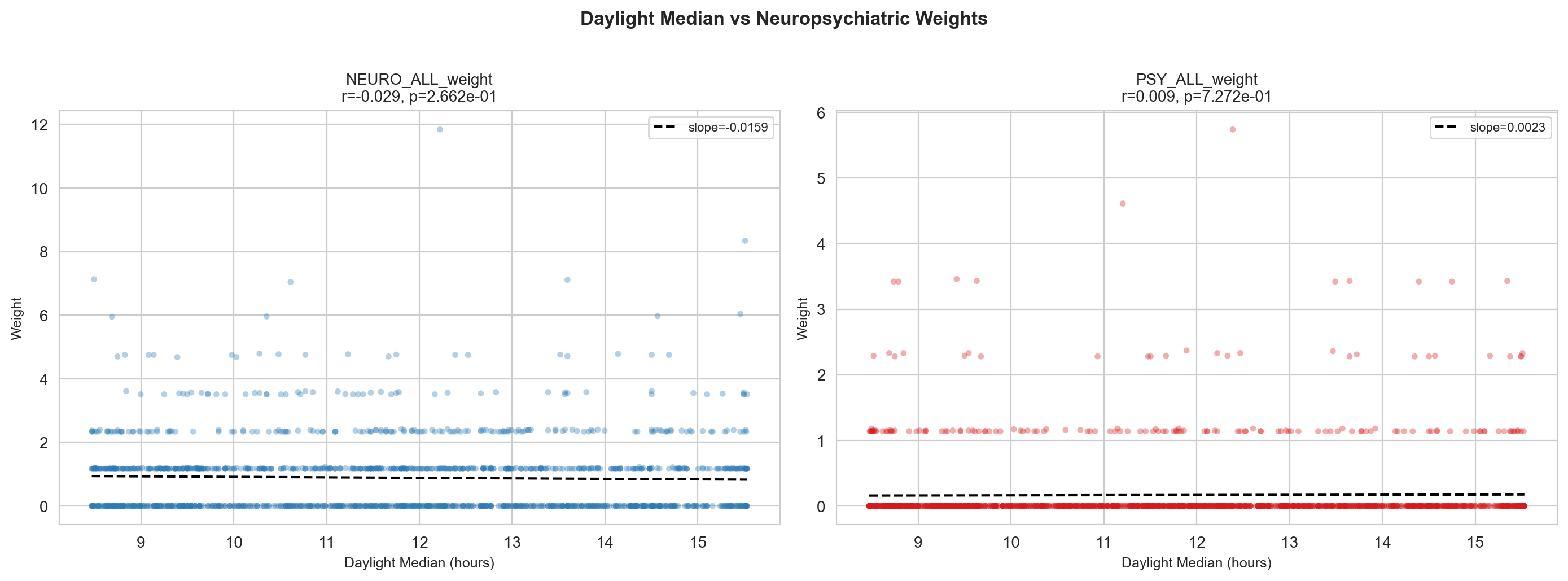

Supplement: Supplementary file 1 [file jcm-15-03030-s001.zip › Figure_S12_daylight_vs_weights.png]

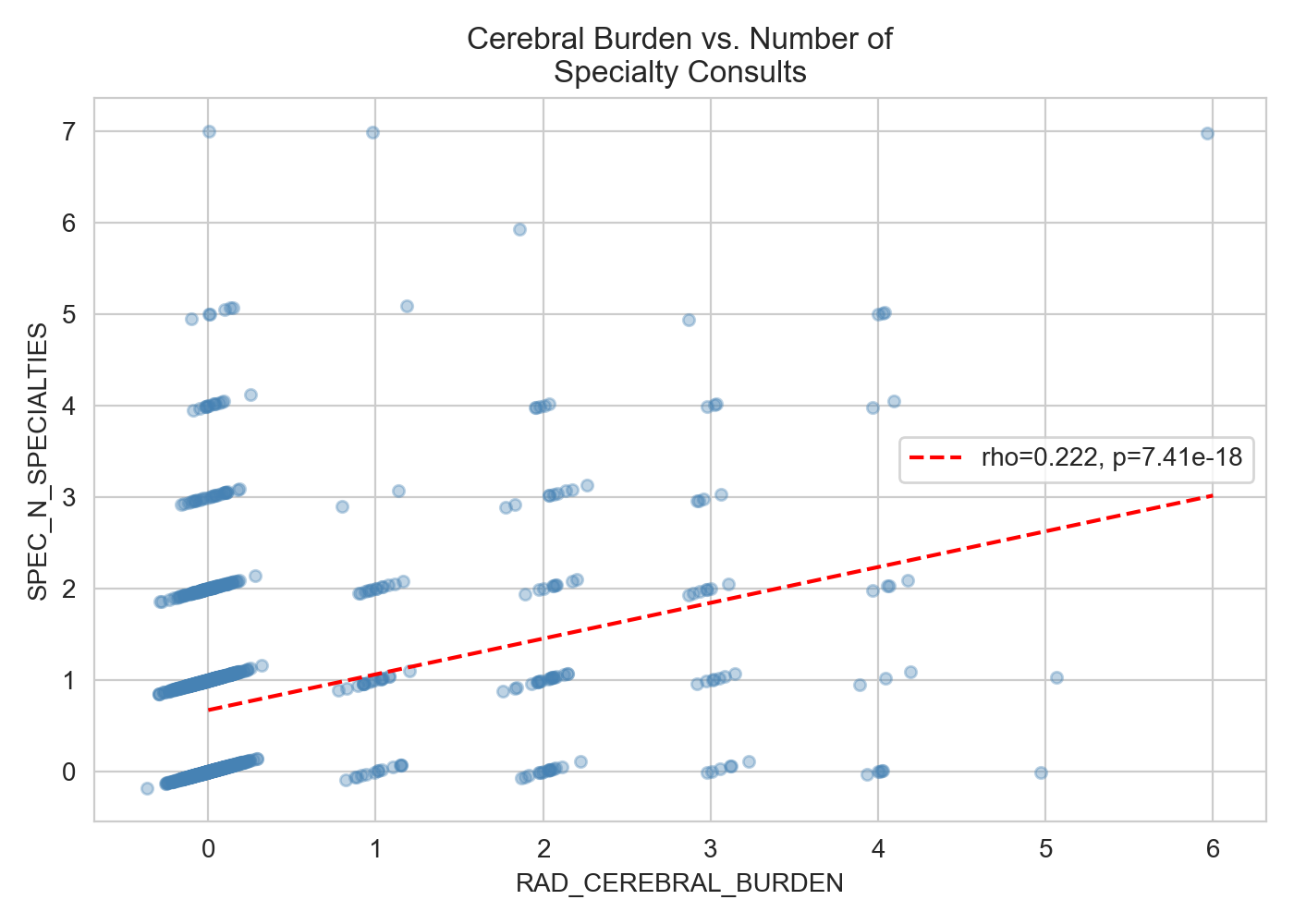

Supplement: Supplementary file 1 [file jcm-15-03030-s001.zip › Figure_S13_burden_vs_specialties.png]

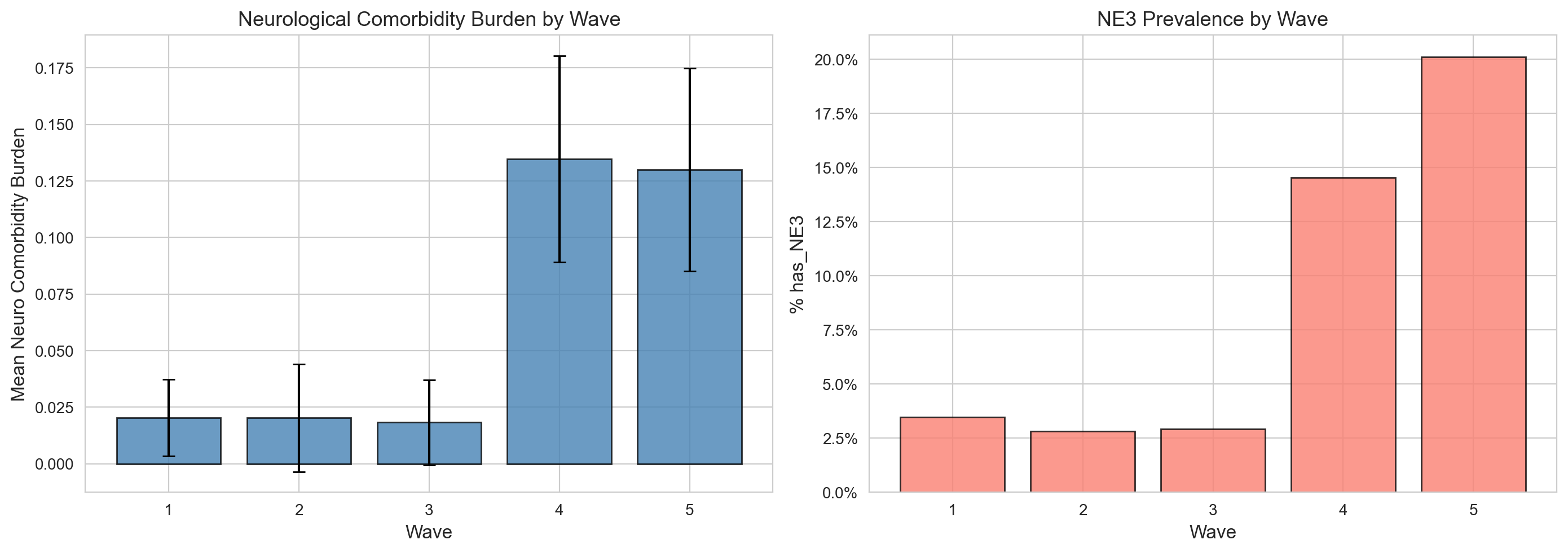

Supplement: Supplementary file 1 [file jcm-15-03030-s001.zip › Figure_S14_neuro_burden_by_wave.png]

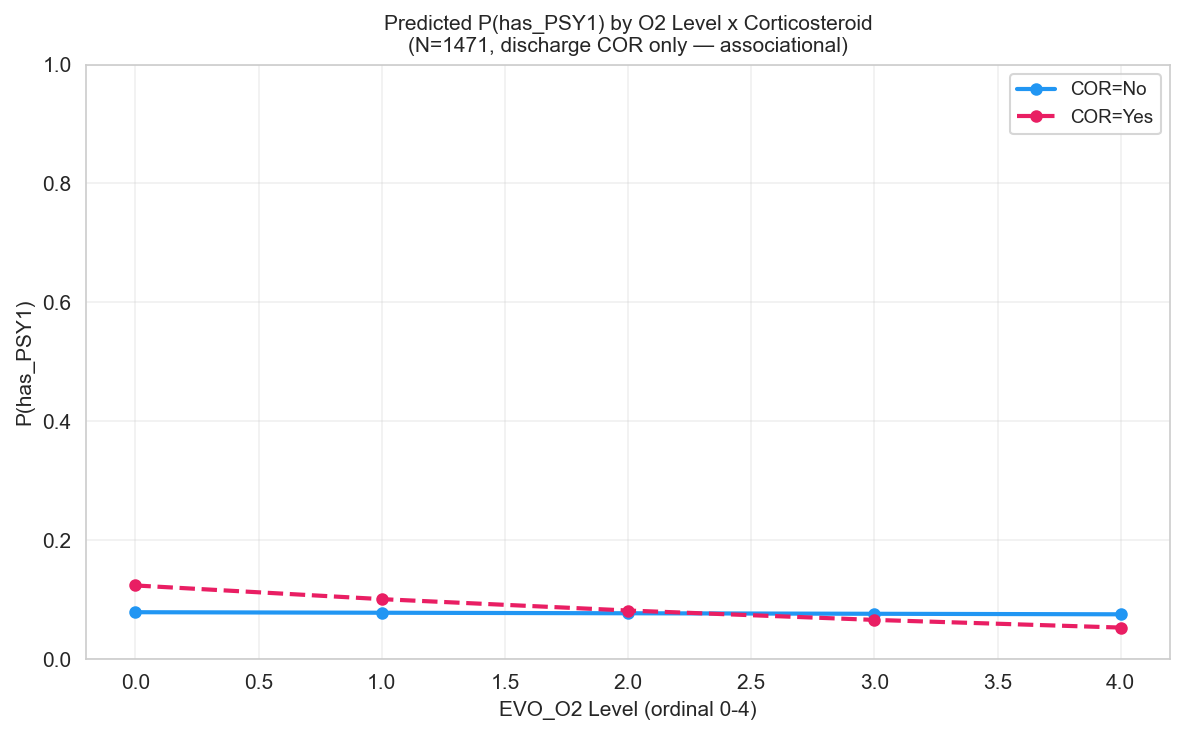

Supplement: Supplementary file 1 [file jcm-15-03030-s001.zip › Figure_S15_corticosteroid_O2.png]

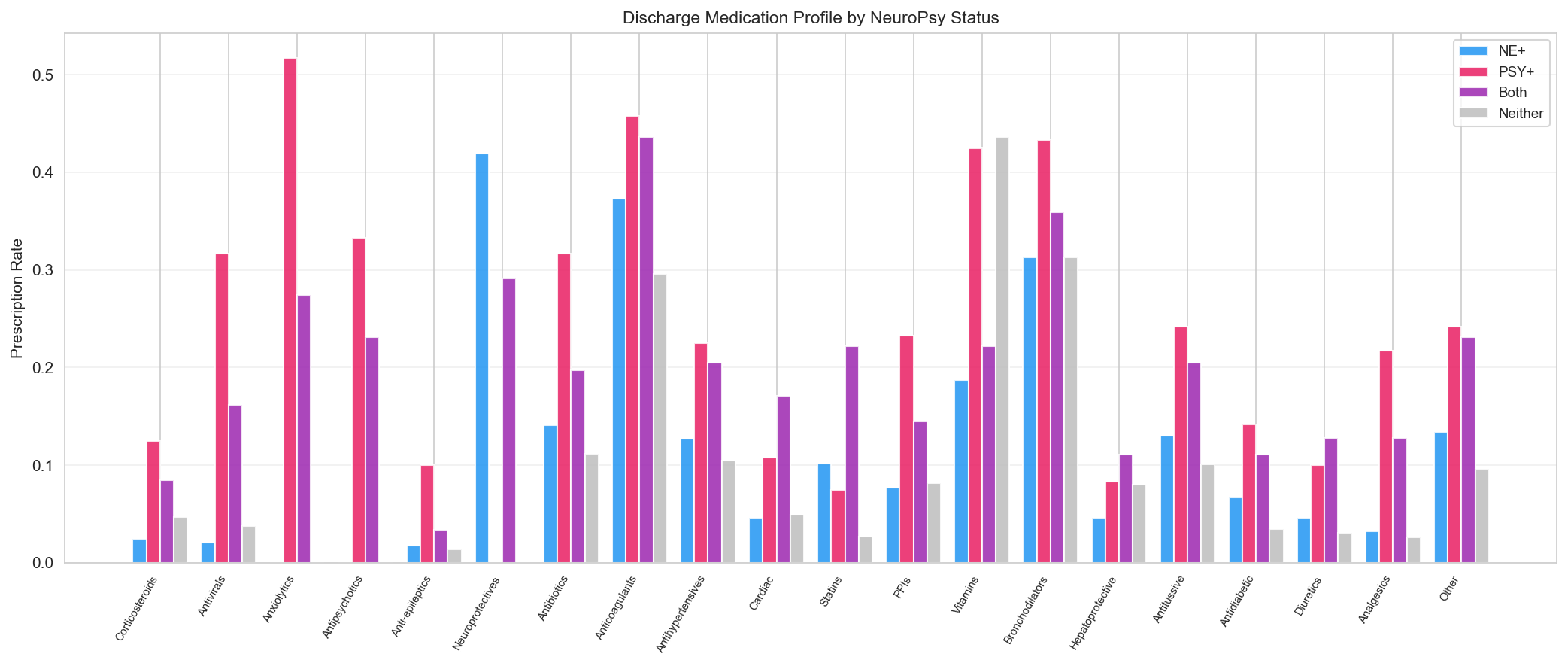

Supplement: Supplementary file 1 [file jcm-15-03030-s001.zip › Figure_S16_med_profile_neuropsy.png]

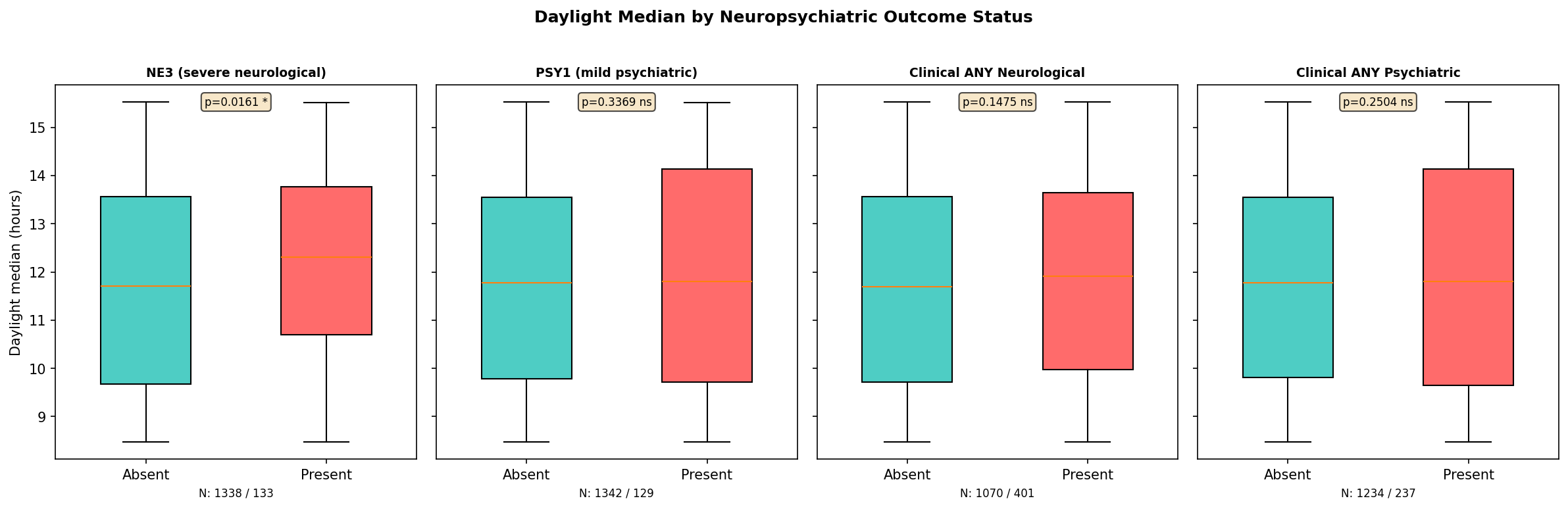

Supplement: Supplementary file 1 [file jcm-15-03030-s001.zip › Figure_S17_daylight_boxplot.png]

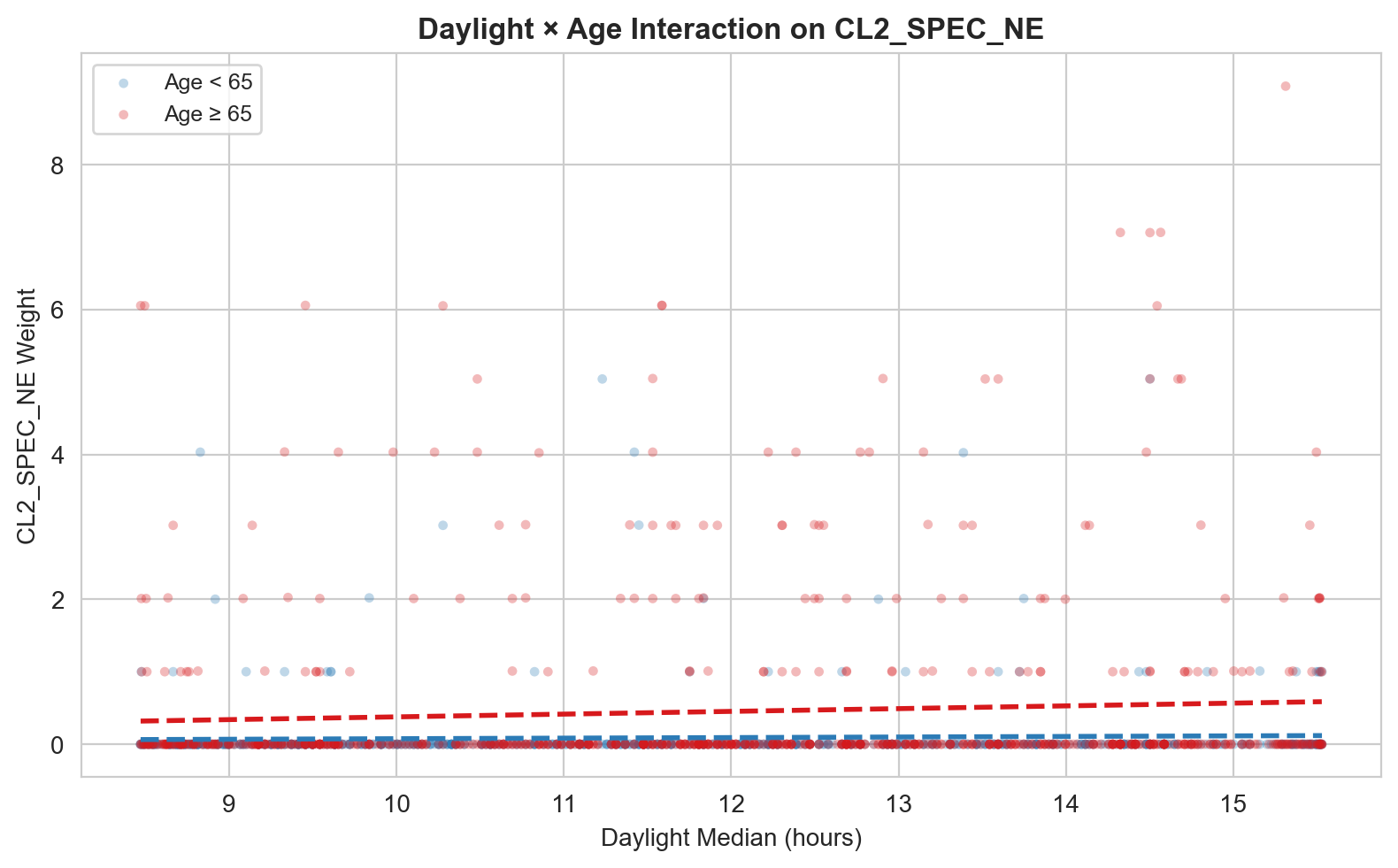

Supplement: Supplementary file 1 [file jcm-15-03030-s001.zip › Figure_S18_daylight_age_interaction.png]

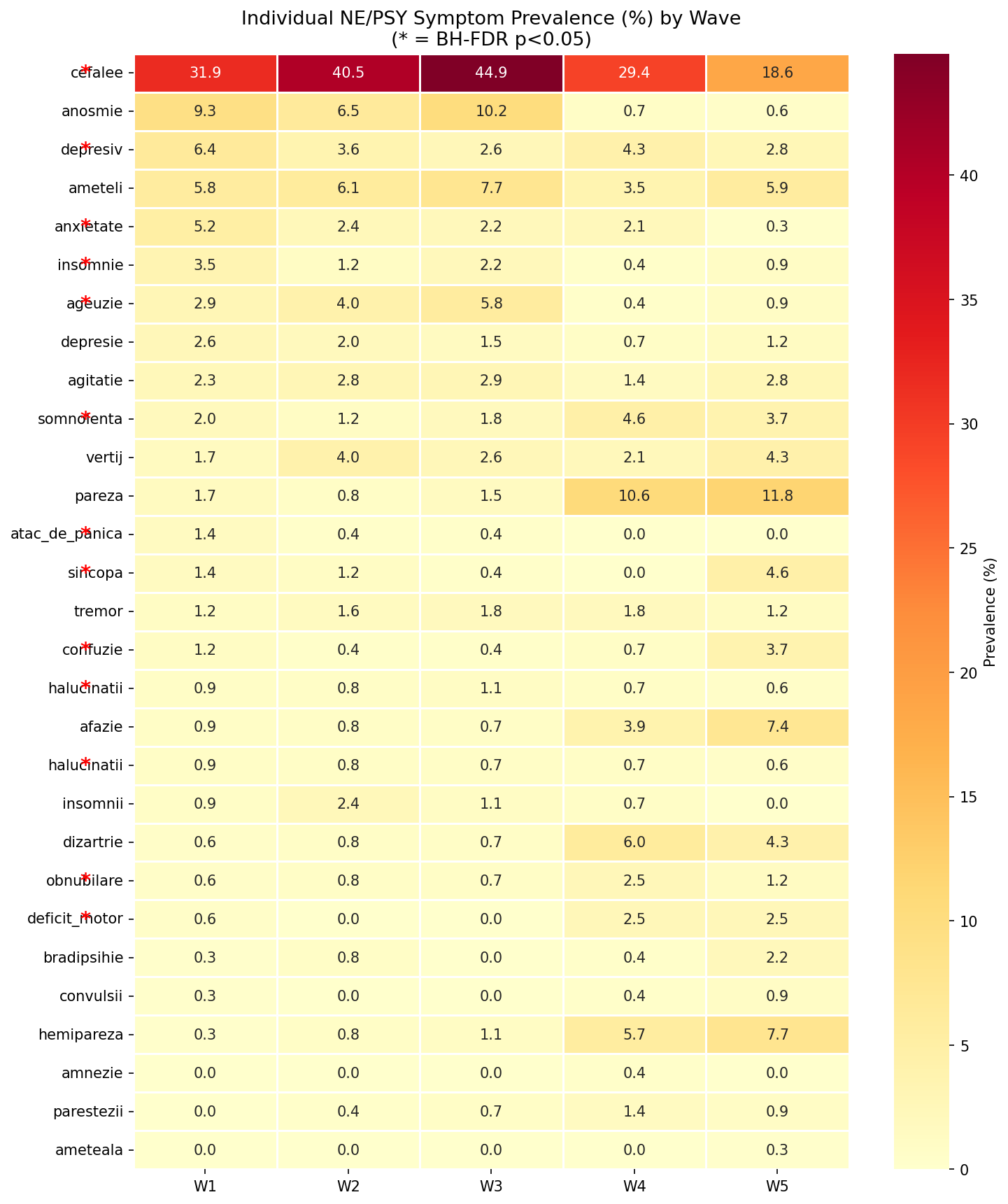

Supplement: Supplementary file 1 [file jcm-15-03030-s001.zip › Figure_S1_symptom_heatmap.png]

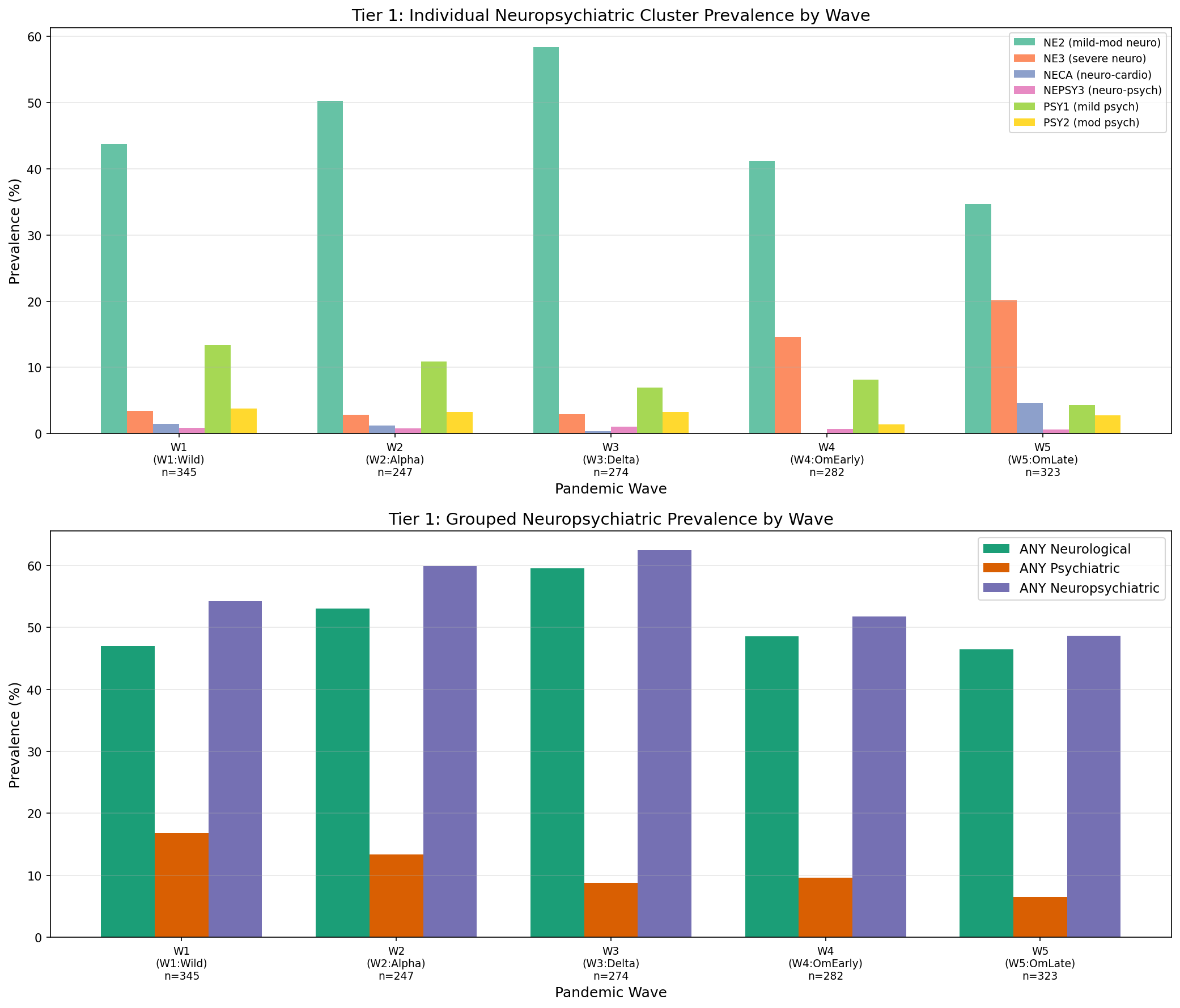

Supplement: Supplementary file 1 [file jcm-15-03030-s001.zip › Figure_S2_tier1_cluster_barplot.png]

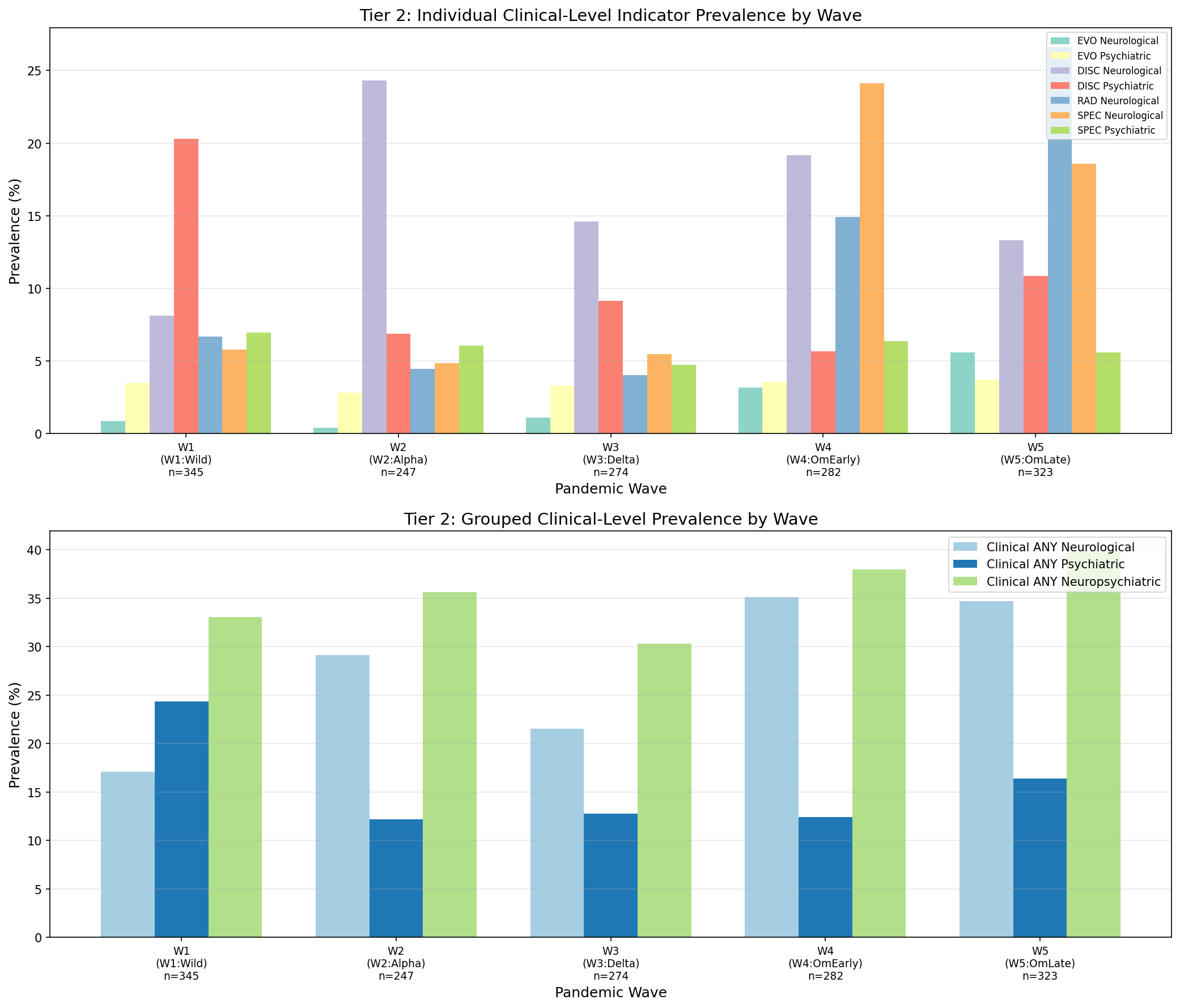

Supplement: Supplementary file 1 [file jcm-15-03030-s001.zip › Figure_S3_tier2_clinical_barplot.png]

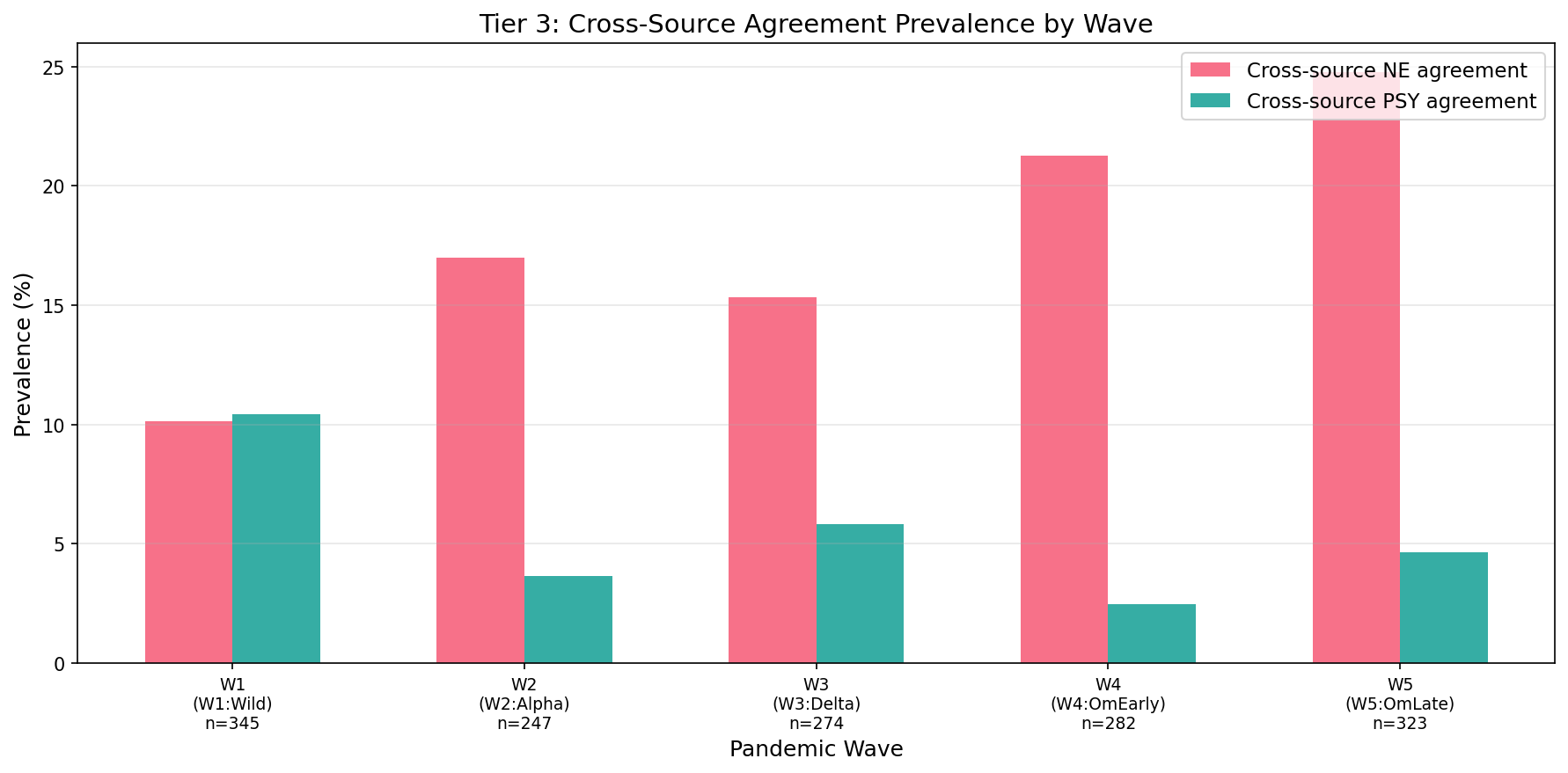

Supplement: Supplementary file 1 [file jcm-15-03030-s001.zip › Figure_S4_tier3_crosssource_barplot.png]

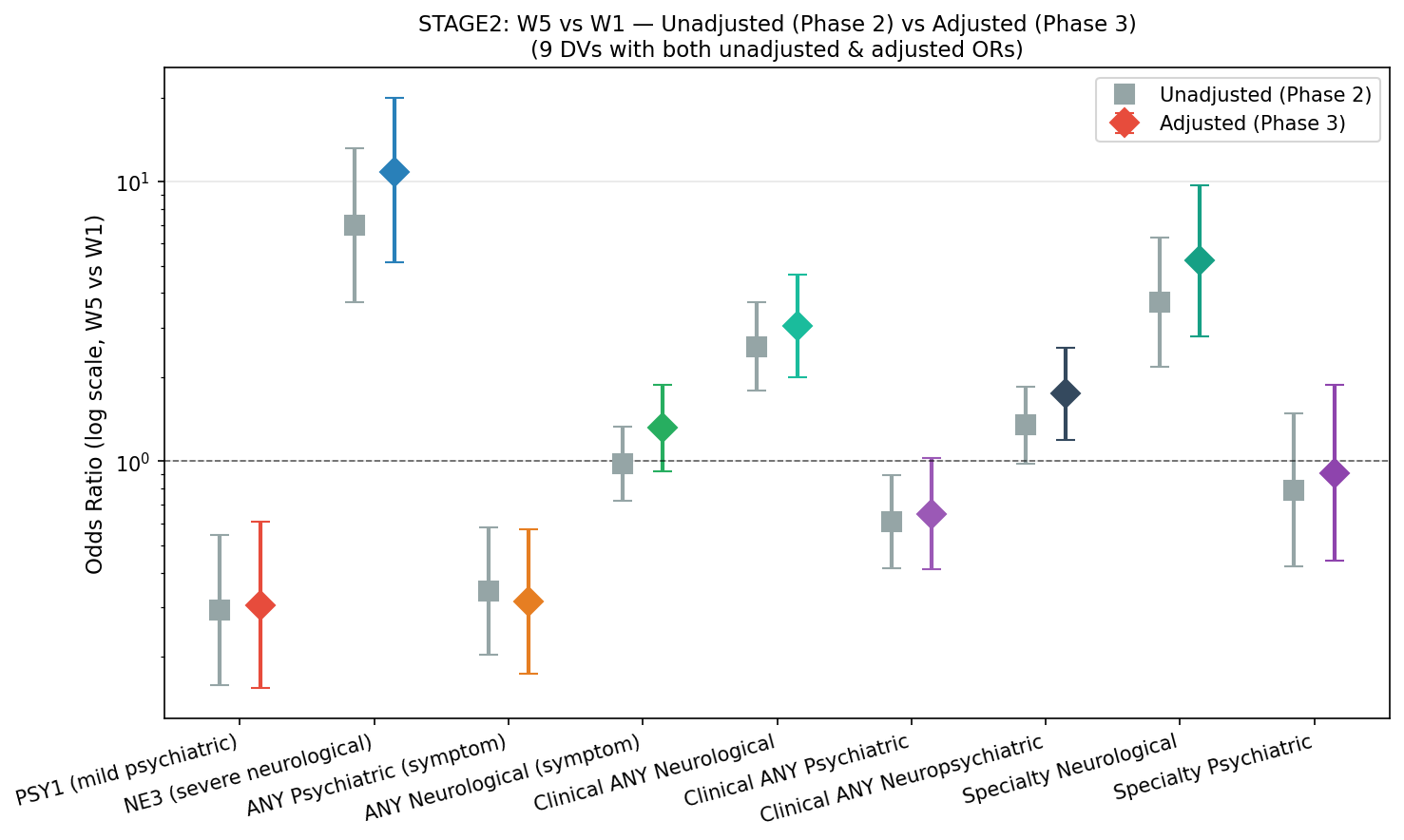

Supplement: Supplementary file 1 [file jcm-15-03030-s001.zip › Figure_S5_unadj_vs_adj.png]

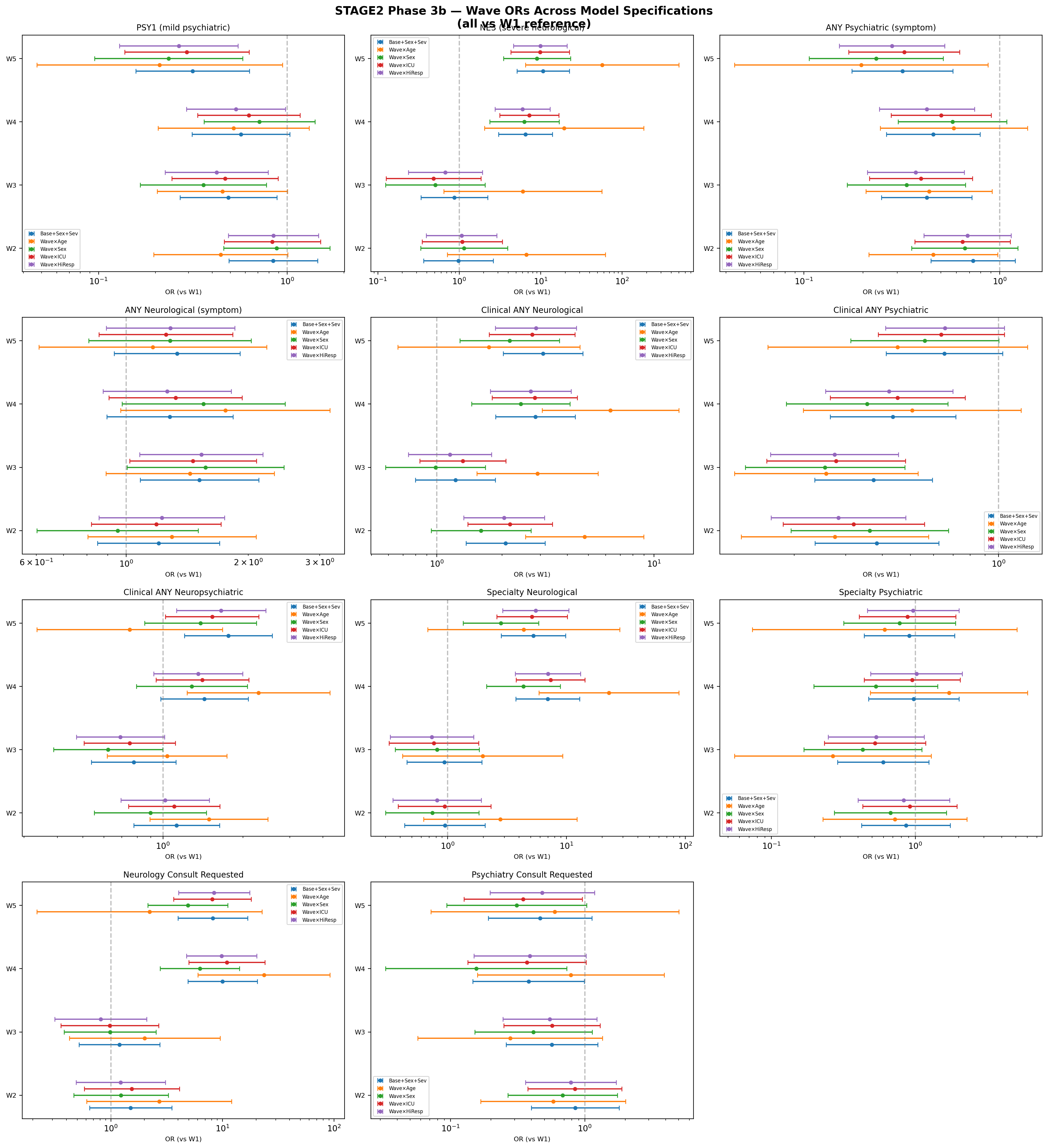

Supplement: Supplementary file 1 [file jcm-15-03030-s001.zip › Figure_S6_sex_interaction_forest.png]

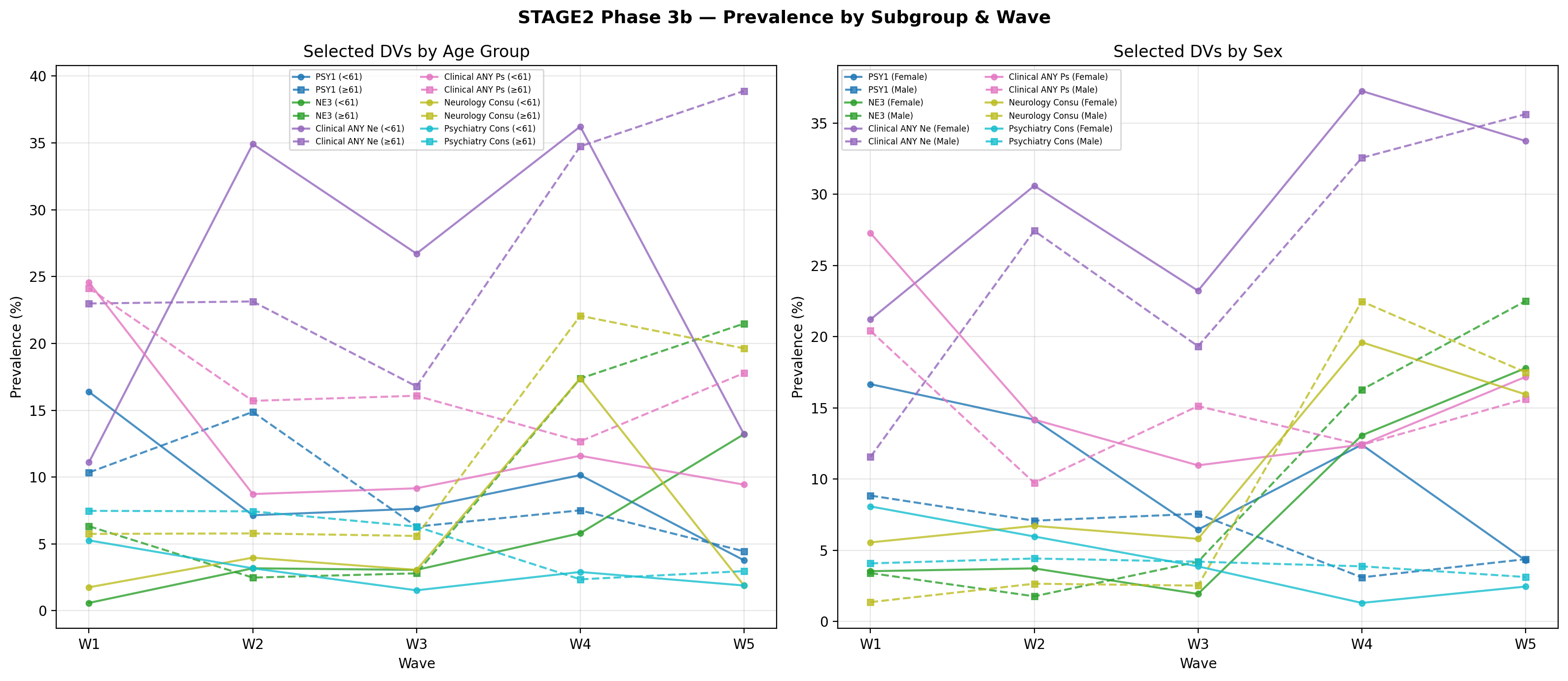

Supplement: Supplementary file 1 [file jcm-15-03030-s001.zip › Figure_S7_sex_subgroup_prevalence.png]

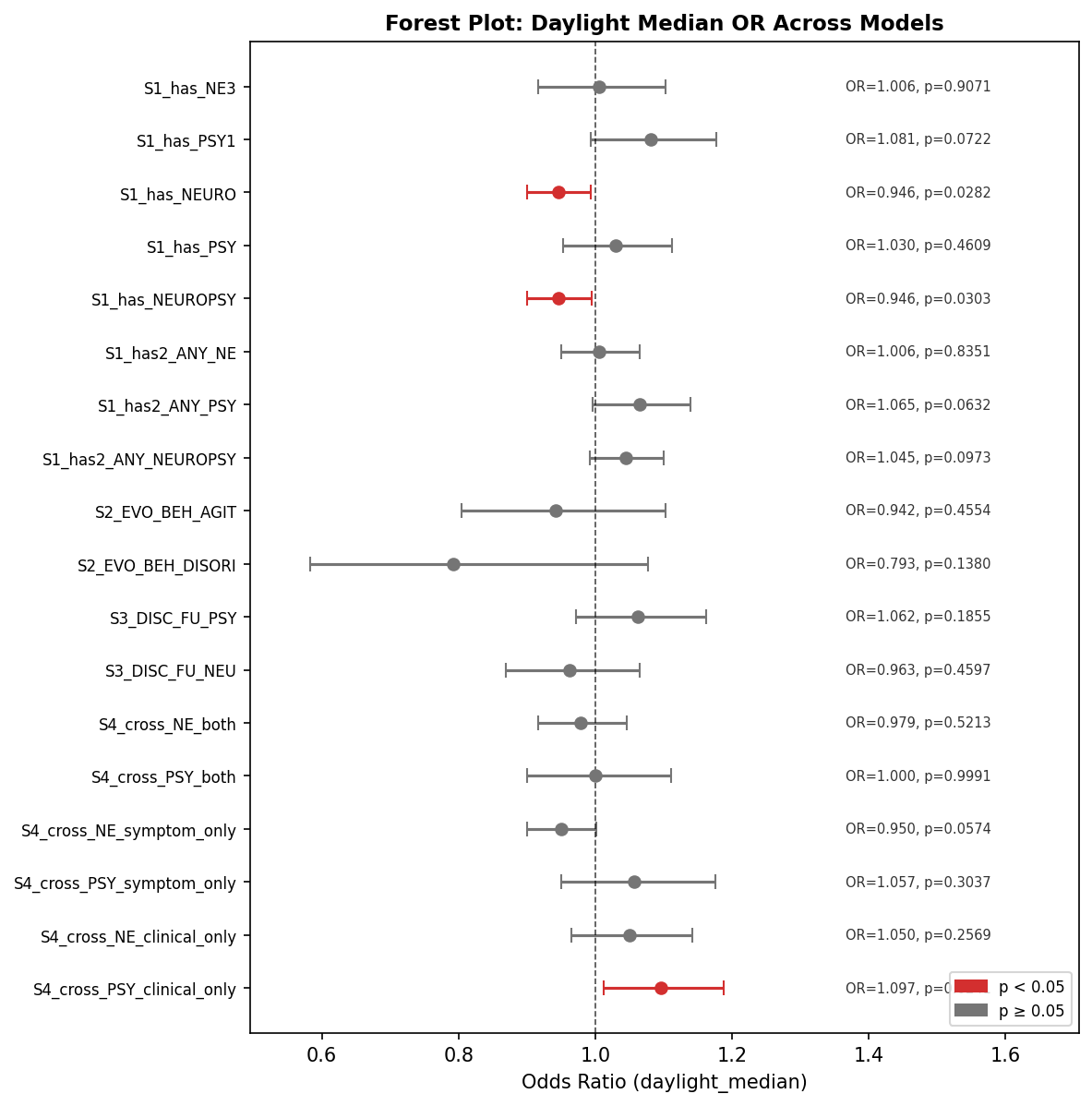

Supplement: Supplementary file 1 [file jcm-15-03030-s001.zip › Figure_S8_daylight_OR.png]

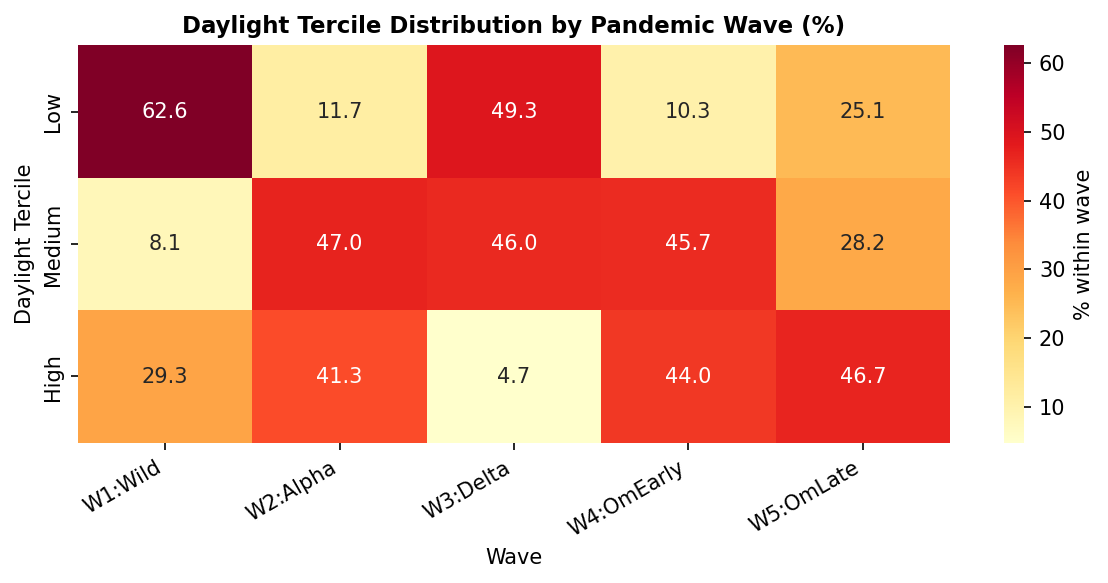

Supplement: Supplementary file 1 [file jcm-15-03030-s001.zip › Figure_S9_daylight_tercile_wave.png]
